# Mitochondria and Lipid Defects in Hereditary Progranulin-Related Frontotemporal Dementia

**DOI:** 10.3390/cells15030276

**Published:** 2026-02-01

**Authors:** Jon Ondaro, Jose Luis Zúñiga-Elizari, Mónica Zufiría, Maddi Garciandia-Arcelus, Ángela Sánchez Molleda, Miren Zulaica, Miguel Lafarga, Javier Riancho, Adolfo López de Munaín, Fermin Moreno, Francisco Javier Gil-Bea, Gorka Gerenu

**Affiliations:** 1 Department of Neuroscience, Biogipuzkoa Health Research Institute (IIS Biogipuzkoa), 20014 San Sebastian, Spain; j.ondaro@gmail.com (J.O.); joseluis.zunigaelizari@bio-gipuzkoa.eus (J.L.Z.-E.); monicazufiria@gmail.com (M.Z.); maddigartz@gmail.com (M.G.-A.); mirenzulaika@hotmail.com (M.Z.); adolfojose.lopezdemunainarregui@osakidetza.eus (A.L.d.M.); fermin.morenoizco@osakidetza.eus (F.M.); 2 Center for Biomedical Research of Neurodegenerative Diseases (CIBERNED), 28029 Madrid, Spain; lafargam@unican.es (M.L.); javier.riancho@unican.es (J.R.); 3 Miaker Developments SL, Miaker Bioassays, 20014 San Sebastián, Spain; 4 Department of Anatomy and Cell Biology, University of Cantabria, 39011 Santander, Spain; 5 Hospital General Sierrallana-IDIVAL, 39300 Torrelavega, Spain; 6 Department of Medicine and Psychiatry, University of Cantabria, 39005 Santander, Spain; 7 Donostia University Hospital, 20014 San Sebastian, Spain; 8 IKERBASQUE Basque Foundation for Science, 48009 Bilbao, Spain; 9 Faculty of Health Sciences, Public University of Navarra, 31008 Pamplona, Spain; 10 Department of Physiology, Faculty of Medicine and Nursing, University of Basque Country (UPV-EHU), 48940 Leioa, Spain

**Keywords:** lysosome, mitochondria, progranulin, GRN, frontotemporal dementia, FTD, lipid droplets, neurodegeneration

## Abstract

**Highlights:**

**What are the main findings?**

- FTD GRN-mutant fibroblasts replicate lysosome-related features observed in FTD patients.
- Fibroblasts from heterozygous GRN c.709-1G>A mutation carriers show altered mitochondrial function.
- Fibroblasts from heterozygous GRN c.709-1G>A mutation carriers exhibit disrupted lipid metabolism.

**What are the implications of the main findings?**

- FTD GRN-mutant fibroblasts provide a useful initial system to investigate lysosomal dysfunction and associated metabolic alterations relevant to FTD.
- These metabolic changes suggest that pathways related to mitochondrial and lipid homeostasis may represent areas of interest for future research.

**Abstract:**

Frontotemporal dementia (FTD) is a neurodegenerative disorder predominantly affecting individuals under 65 years of age, characterized by significant behavioral and language disabilities. Despite extensive research efforts, effective treatments for FTD remain elusive. Familial cases of FTD have been linked to genetic mutations in several key genes, among these, mutations in granulin (*GRN*) account for 5–20% of cases, leading to haploinsufficiency of progranulin (PGRN), a multifunctional glycoprotein. This study investigates the cellular pathology associated with *GRN* insufficiency by using fibroblasts derived from FTD patients carrying the c.709-1G>A *GRN* mutation (FTD-GRN). These fibroblasts exhibited pathological hallmarks of FTD, including lysosomes, autophagosomes, and lipofuscin accumulation, mirroring observations in affected patient tissues. Notably, we report mitochondrial abnormalities, characterized by mitochondrial swelling which is associated with decreased mitochondrial respiration, and lipid droplet accumulation, reflecting altered lipid metabolism. Experimental supplementation with recombinant human progranulin (rhPGRN) was associated with recovery of lysosomal acidification and attenuation of mitochondrial and lipid abnormalities in vitro. This study reveals that *GRN* haploinsufficiency induces mitochondrial and lipid dysfunctions, suggesting that these pathways may contribute to FTD-GRN pathogenesis and could be of interest for therapeutic development.

## 1. Introduction

Frontotemporal dementia (FTD) is a neurodegenerative disorder and the second leading cause of dementia in individuals under 65 years of age, characterized by progressive impairments in behavior and language [1]. Despite ongoing progress, there are no effective treatments for FTD. Age is considered the most important risk factor for FTD and the majority of cases are sporadic, meaning there is no clear family history. However, approximately 40% of patients of FTD do have a family history of dementia, suggesting a significant genetic component in the development of the disease [2]. Mutations in several genes including *MAPT*, *GRN*, *FUS*, *VCP*, and *C9orf72* are known to cause familial frontotemporal dementia (fFTD). Among them, mutations in the granulin (*GRN*) gene account for 5–20% of the total fFTD cases [3]. The heterozygous mutations lead to a haploinsufficiency of its encoding protein progranulin (PGRN), resulting in a production of a truncated PGRN protein [4] and triggering neurodegeneration. One of these FTD-causing mutations was discovered 15 years ago in a cluster of Basque families carrying a unique ancestral genetic substitution (c.709-1G>A) in the *GRN* gene [5]. This mutation is representative of the majority of *GRN*-associated FTD cases, as most GRN mutations result in loss of function and lead to disease through *GRN* haploinsufficiency.

PGRN is a secreted glycoprotein composed of 7.5 GRN segments (granulins A-G and paragranulin), that play significant roles in several cellular processes [6]. Although the precise function of PGRN is not yet fully understood, accumulating evidence suggests its critical role in lysosomal function [7,8,9]. Intracellularly, full-length PGRN predominantly localizes within the lysosome, where it undergoes proteolytic cleavage by lysosomal proteases, resulting in the generation of individual GRN peptides [10]. These peptides are believed to regulate lysosomal enzymes such as cathepsin D [11] and glucocerebrosidase [12,13]. Dysfunction of lysosomal enzymes leads to impaired lysosomal function and is associated with lysosomal storage disorders (LSDs). Homozygous *GRN* mutations, resulting in complete loss of PGRN, cause neuronal ceroid lipofuscinosis (NCL), a type of LSD. NCL shares pathological features with FTD due to *GRN* mutation (FTD-GRN), including the accumulation of lipofuscin granules [14] and dysfunctional lysosomes [15]. Consequently, lysosomal dysfunction is widely recognized as a key contributor to neurodegeneration in FTD-GRN [9].

The lysosome is a dynamic organelle that coordinates waste recycling with nutrient sensing and metabolic regulation, ensuring cellular homeostasis and survival. To fulfill this role, the lysosome interacts with other cellular structures exchanging contents and information by establishing membrane contact sites [16].

As observed in some pathological conditions, lysosomal dysfunction induces alterations in mitochondria and lipid metabolism [17,18,19], reflecting the close relationship between these mechanisms. Under physiological conditions, their coordination is essential for cellular adaptation, reconfiguration of nutrient utilization, and maintenance of metabolic balance during periods of energy scarcity. Lysosomal dysfunction can, in some cases, mimic this state, inducing a starvation (STV)-like metabolism. This reconfiguration involves a shift away from glycolysis towards mitochondrial β-oxidation of fatty acids (FAs) derived from triacylglycerides (TAG) or cholesterol esters stored within lipid droplets (LDs) [20]. During energy stress, macroautophagy is enhanced to recycle cellular components and supply the cell with essential materials to prioritize vital biological processes [21]. Within the lysosome, membrane lipids are broken down into FAs and released. These FAs can serve as a source of energy production for the mitochondria or may be re-esterified and packaged into new LDs in the endoplasmic reticulum, leading to a paradoxical increase in LDs [22,23]. This mechanism helps to manage the excessive influx of FAs into the mitochondria and prevents lipotoxicity [24,25].

Based on these considerations, we hypothesized that *GRN* haploinsufficiency associated with FTD-GRN, may lead to secondary alterations in organelles, such as mitochondria and lipid trafficking, ultimately causing detrimental metabolic consequences. To this end, we used human fibroblasts derived from FTD-GRN patients carrying the c.709-1G>A mutation.

## 2. Materials and Methods

### 2.1. Human Samples

Six skin biopsies were analyzed, including samples from *GRN* c.709-1G>A mutation carriers (GRN+/−) and healthy control (CTL) relatives. All patients were diagnosed and treated at Donostia University Hospital according to established consensus criteria [26]. Biopsies and genetic analyses were performed with written informed consent and approval from the Ethics Committee of the Government of the Basque Country.

### 2.2. Cell Culture Conditions

Primary fibroblasts from CTL donors and *GRN* c.709-1G>A mutation carriers were cultured in cell medium (CM), DMEM (Gibco, Thermo Fisher Scientific, Paisley, UK) supplemented with FBS (Gibco), GlutaMAX (Thermo Fisher Scientific), and penicillin/streptomycin (Gibco) at 37 °C and 5% CO_2_. STV conditions were induced using supplemented EBSS for 6 h. Cells were treated with chloroquine (CLQ) (Sigma-Aldrich, Darmstadt, Germany), bafilomycin A1 (BafA1) (Selleckchem, Munich, Germany) or recombinant human progranulin (rhPGRN) (R&D Systems Europe Ltd., Abingdon, Oxfordshire, UK) at the indicated concentrations and times. The vehicle was referred to as non-treated (NT).

### 2.3. mRNA Expression/Processing Analysis

Total RNA was isolated using the RNeasy Mini Kit (Qiagen, Hilden, Germany) and reverse-transcribed with SuperScript VILO (Thermo Fisher Scientific). qRT-PCR was performed using SYBR Green on a CFX384 Touch Real-Time PCR Detection System (Bio-Rad Europe GmbH, Basel, Switzerland), with GAPDH as the reference gene and relative expression calculated by the 2^−ΔΔCt^ method.

### 2.4. Protein Extraction and Western Blotting

Primary fibroblasts were lysed, and proteins were separated by SDS–PAGE and transferred to nitrocellulose membranes. Membranes were probed with specific primary antibodies and HRP-conjugated or fluorescent secondary antibodies. Signals were detected using the iBright imaging system (Thermo Fisher Scientific), quantified with Image Studio Lite (LI-COR Biosciences, Lincoln, NE, USA), and normalized to β-tubulin.

### 2.5. Immunofluorescence

Primary fibroblasts were cultured on Ibidi slides, fixed with paraformaldehyde, and blocked/permeabilized with Bovine serum albumin (BSA) and Triton X-100. Cells were incubated with primary antibodies against LC3B, LAMP-1, or TOM20, followed by Alexa Fluor–conjugated secondary antibodies and DAPI nuclear staining. Images were acquired using a Zeiss LSM 900 confocal microscope (Carl Zeiss, Jena, Germany), and image and colocalization analyses were performed with ImageJ (version 1.54, National Institutes of Health, Bethesda, MD, USA).

### 2.6. Live Cell Imaging

Lysosomal activity was assessed using LysoTracker™ Red (Thermo Fisher Scientific)–labeled fibroblasts imaged by confocal microscopy. Fatty acid pulse–chase assays were performed using BODIPY 558/568 C12 (Red-C12) (Life Technologies Europe BV, Bleiswijk, The Netherlands) to track lipid incorporation into LDs and trafficking to mitochondria, with LDs labeled by BODIPY 493/503 (BD493) (Life Technologies) and mitochondria by MitoTracker™ Green (Invitrogen, Thermo Fisher Scientific, Breda, The Netherlands). Live-cell imaging was conducted on a Zeiss LSM 900 confocal microscope (Carl Zeiss), with cells maintained at 37 °C in a controlled CO_2_ atmosphere. Image quantification of LDs, lysosomes, and FAs distribution was performed using ZEN 3.1 Blue (Carl Zeiss), ImageJ (NIH), and Cellpose-based cell segmentation [27].

### 2.7. Seahorse XF-96 Metabolic Flux Analysis

Oxygen consumption rates (OCRs) were measured using a Seahorse XF-96 Analyzer (Agilent Technologies GmbH, Waldbronn, Germany). Primary fibroblasts were seeded in XF-96 plates and, when indicated, exposed to STV conditions prior to analysis. Mitochondrial function was assessed using the XF Cell Mito Stress Test with sequential injections of oligomycin, FCCP, and rotenone/antimycin A to calculate basal respiration, ATP production, and maximal and spare respiratory capacities.

Fatty acid oxidation (FAO) dependency was evaluated using the Seahorse FAO assay with palmitate-BSA, followed by sequential injections of etomoxir and mitochondrial inhibitors. OCR values were normalized to cell number, and metabolic parameters were calculated using Wave Agilent Software 2.4.2 (Agilent Technologies).

### 2.8. Transmission Electron Microscopy

Primary fibroblasts were fixed in 3% glutaraldehyde and washed before processing for transmission electron microscopy (TEM) at the Principe Felipe Research Center. Ultrathin sections (70 nm) were imaged at the Core Facility for Polymer Characterization (UPV/EHU, San Sebastian, Spain). Quantification of lysosomes, autophagosomes, fingerprint structures, LDs, and mitochondrial area was performed in 10–15 cells per sample and expressed relative to total cell area. Mitochondrial cristae were analyzed following established methods [28].

### 2.9. Statistical Methods

Data are shown as mean ± SEM from at least three independent experiments. Statistical analyses were performed using GraphPad Prism 10.0.0 (GraphPad Software, San Diego, CA, USA). Two-group comparisons used the Mann–Whitney U test, while multi-factor comparisons were analyzed by two-way ANOVA with Tukey’s post hoc test. Significance is indicated as ns (*p* > 0.05), * *p* < 0.05, ** *p* < 0.01, *** *p* < 0.001, **** *p* < 0.0001.

## 3. Results

### 3.1. Skin Fibroblasts from FTD-GRN Patients Reproduce the Neuropathological Hallmarks Observed in FTD Brains

**Figure 1:**
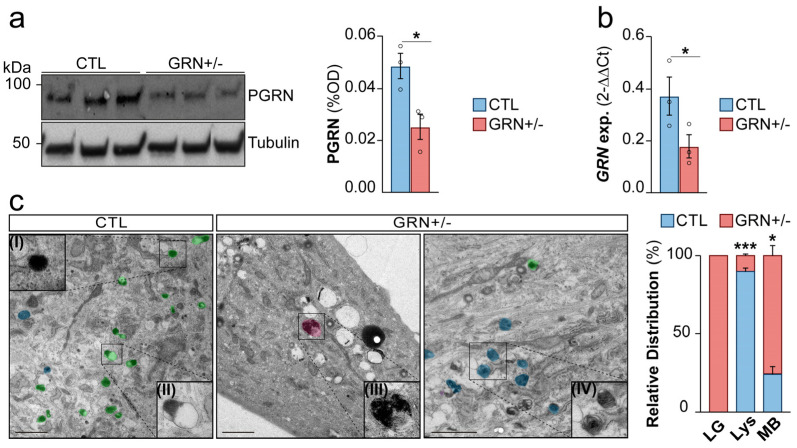
Pathological Features in Fibroblasts from Heterozygous *GRN*c.709-1G>A Mutation Carriers Resemble FTD Pathology. (**a**) PGRN protein levels assessed by Western blotting in primary human fibroblasts. Quantifications were normalized to the loading control (β-Tubulin). (**b**) PGRN mRNA expression analyzed by qPCR in primary human fibroblasts. (**c**) Representative TEM images of primary human fibroblasts showing lysosomes (green), multilamellar bodies (blue), and lipofuscin bodies (pink). Insets: (I) primary lysosome (electron-dense and homogeneous) and (II) secondary lysosome (heterogeneous structure). (III) Lipofuscin body (IV) multilamellar body. Scale bar: 2 μm. Optical density (OD), Lipofuscin granule (LG), Lysosome (Lys), Multilamellar bodies (MB). Data are shown as mean ± SEM of independent cell lines (*n* = 3 per group) from one experiment. Each data point represents the mean of 1–8 technical replicates for a single line depending on the assay. The experiment was repeated independently three times with similar results. Statistical significance is denoted as follows: * *p* < 0.05 and *** *p* < 0.001.

Heterozygous pathogenic GRN variants cause an approximately 50% reduction in both PGRN mRNA and protein levels [29,30]. This feature was reproduced in GRN+/− fibroblasts, showing significantly reduced PGRN protein levels (*p* < 0.05) (Figure 1a, uncropped images in Figure S1) and mRNA levels when compared with CTL fibroblasts (*p* < 0.05) (Figure 1b). Based on this finding, we investigated whether this reduction could induce FTD-like pathology in patient fibroblasts.

FTD-GRN patients also showed an accumulation of lipofuscin granules and lysosomal deposits [14]. These pathological features are typically associated with NCL, a disease caused by the complete absence of PGRN. To explore these pathological features, both CTL and GRN+/− fibroblasts were examined by TEM. GRN+/− fibroblasts showed a prominent accumulation of lysosome-related storage material, with a significantly higher proportion of multilamellar bodies compared to the CTLs (*p* < 0.05) (Figure 1c, detail in Figures S2–S4). Furthermore, dense lipofuscin granules, which were not present in CTLs, were observed in GRN+/− fibroblasts (Figure 1c, detail in Figures S2–S4). Additionally, the number of electron-dense lysosomes was reduced in the GRN+/− fibroblasts in comparison to CTLs (*p* < 0.05) (Figure 1c, detail in Figures S2–S4). We then sought to quantitatively validate these putative lysosomal defects and to investigate whether they were a direct consequence of PGRN deficiency.

### 3.2. Skin Fibroblasts from FTD-GRN Patients Exhibit Impaired Lysosomal Function

**Figure 2:**
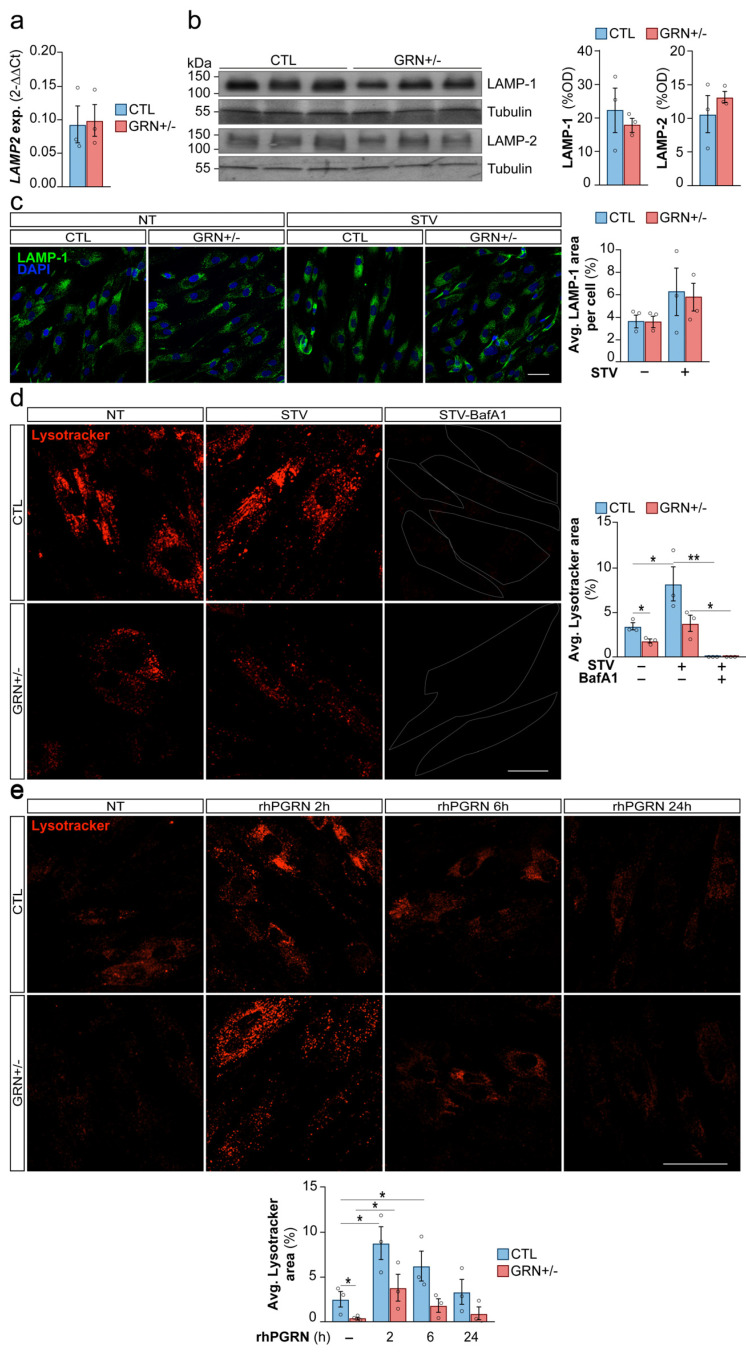
Impaired Lysosomal Function in Fibroblasts from Heterozygous *GRN*c.709-1G>A Mutation Carriers. (**a**) mRNA expression of LAMP2 assessed by qPCR in primary human fibroblasts. (**b**) Protein levels of LAMP-1 and LAMP-2 analyzed by Western blotting in primary human fibroblasts. Quantifications were normalized to the loading control (β-Tubulin). (**c**) Representative images of LAMP-1 (green) and DAPI (blue) staining in primary human fibroblasts cultured with or without a 6 h pre-assay period under STV conditions. Scale bar: 50 µm. (**d**) Representative images and quantification of LysoTracker™ Red DND-99 staining in primary human fibroblasts. Cells were cultured with or without a 6 h pre-assay period under STV conditions, followed by 2 h of incubation with BafA1 (100 nM) prior to live imaging. LysoTracker™ Red DND-99 staining was used to visualize lysosomal compartments. Scale bar: 40 µm. (**e**) Representative images and quantification of LysoTracker™ Red DND-99 staining in primary human fibroblasts treated with rhPGRN (500 ng/mL) at different time points (2, 6, and 24 h). LysoTracker™ Red DND-99 staining was used to visualize lysosomal compartments. Scale bar: 71 µm. Optical density (OD). Data are shown as mean ± SEM of independent cell lines (*n* = 3 per group) from one experiment. Each data point represents the mean of 1–8 technical replicates for a single line depending on the assay. The experiment was repeated independently three times with similar results. Statistical significance is indicated as follows: * *p* < 0.05 and ** *p* < 0.01.

As the accumulation of multilamellar bodies indicates lysosomal dysfunction, and this is consistently observed in animal and cellular models of PGRN deficiency [31,32,33], we investigated lysosomal function in GRN+/− fibroblasts. First, we assessed lysosomal load by measuring the protein levels of two lysosomal structural proteins, LAMP-1 and LAMP-2, and the mRNA levels of LAMP2, and found no differences between GRN+/− and CTL fibroblasts (Figure 2a,b, uncropped images in Figure S5). Furthermore, immunofluorescence analysis revealed no differences in lysosomal localization between GRN+/− and CTL fibroblasts under both CM and STV conditions. The similar LAMP-1 levels in *GRN* haploinsufficient and CTL fibroblasts under STV condition suggest that these mutant cells retain the ability to synthesize lysosomal structures comparably to non-mutant cells (Figure 2c).

However, we observed a significant reduction in the staining of Lysotracker Red DND-99, a dye used to label acidic organelles in live cells, in the GRN+/− fibroblasts compared to CTL fibroblasts (*p* < 0.05). After six hours of STV, the number of active lysosomes increased as expected in CTL fibroblasts when compared to the CM fibroblasts (*p* < 0.05), but not in GRN+/− fibroblasts. However, in both CTL (*p* < 0.01) and GRN+/− fibroblasts (*p* < 0.05), treatment with BafA1, an inhibitor of the vacuolar H^+^-ATPase, resulted in inhibition of lysosomal function when compared to NT fibroblasts (Figure 2d, uncropped images in Figure S6). This analysis in lysosomal function, revealed that, despite the equal number of lysosomal structures, the functional units are reduced in GRN+/− fibroblasts due to impaired lysosomal acidification, which may explain the observed accumulation of storage material in TEM images. To evaluate whether lysosomal dysfunction is directly linked to insufficient PGRN, we used 500 ng/mL of rhPGRN as a proof-of-concept for protein replacement in our cell model. We assessed Lysotracker Red DND-99 staining at 2, 6, and 24 h to monitor lysosomal acidification. The peak of Lysotracker staining manifested at 2 h post treatment was consistently observed in both CTL and GRN+/− fibroblasts when compared to NT fibroblasts (*p* < 0.05) (Figure 2e, uncropped images in Figure S7). Notably, the 2 h rhPGRN treatment in GRN+/− fibroblasts restored lysosomal function comparable to those observed in untreated CTLs (Figure 2e). This observation supports a strong association between PGRN levels and lysosomal acidification in fibroblasts from FTD-GRN patients.

### 3.3. Skin Fibroblasts from FTD-GRN Patients Display Abnormalities of Autophagy

Lysosomes play a pivotal role in the resolution of the autophagy to facilitate the enzymatic degradation of the cellular cargo. In fact, alterations in autophagic flux have been described in several studies of PGRN deficiency [32,33]. When fibroblasts are cultured under CM, they do not show a significant level of basal autophagic activity. This observation is supported by the low levels of lipidated microtubule-associated protein 1A/1B light chain 3 (LC3B-II), a well-established marker of autophagy-related structures, under CM (Figure S8a). Therefore, these findings suggest that autophagy is not essential under CM conditions in fibroblasts. Consistently, we observed an increase in the level of LC3B-II in fibroblasts after 5 h of lysosomal inhibitor CLQ, and no significant difference between samples derived from CTL individuals and FTD-GRN patients (Figure S8b). Therefore, under CM conditions, it becomes challenging to detect a potential alteration in autophagy due to *GRN* haploinsufficiency.

**Figure 3:**
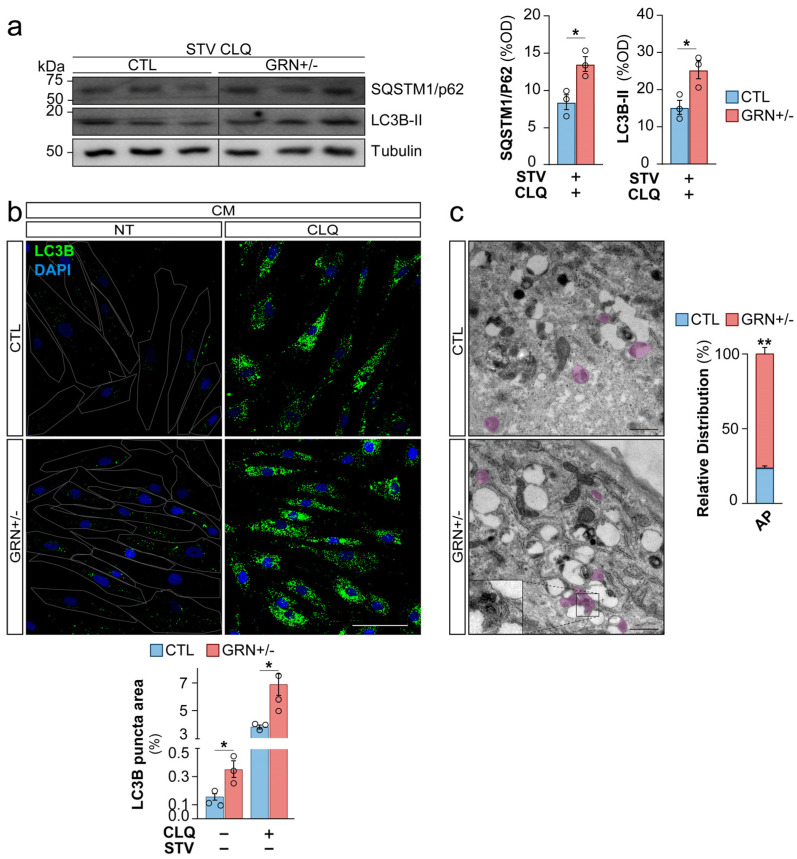
Dysregulated Autophagy in Fibroblasts from *GRN*c.709-1G>A Mutation Carriers. (**a**) Protein levels of LC3B-II and P62 assessed by Western blotting in primary human fibroblasts. Cells were cultured with or without a 6 h STV pre-assay period and treated with CLQ (30 µM) for 5 h. western blotting was performed to assess LC3B-II and P62 protein levels, and quantifications were normalized to the loading control (β-Tubulin). (**b**) Representative images of LC3B puncta (green) and DAPI (blue) staining in primary human fibroblasts. Cells were treated with or without a 5 h pre-assay period with CLQ (30 µM). LC3B puncta (green) staining and DAPI (blue) staining were used to visualize autophagosomes and nuclei, respectively. Scale bar: 100 µm. (**c**) Representative TEM images of primary human fibroblasts, showing autophagosomes (purple). Insets: Autophagosome. Scale bar: 1 µm. Optical density (OD), Autophagosome (AP). Data are shown as mean ± SEM of independent cell lines (*n* = 3 per group) from one experiment. Each data point represents the mean of 1–8 technical replicates for a single line depending on the assay. The experiment was repeated independently three times with similar results. Statistical significance is indicated as follows: * *p* < 0.05 and ** *p* < 0.01.

On the contrary, a prominent activation of autophagosome biogenesis can be detected under STV conditions. Moreover, fibroblasts were cultured in STV medium for 6 h prior to the assay and treated with CLQ to measure the autophagy flux in these cells by measuring LC3B-II levels and sequestosome 1 (P62/SQSTM1), an ubiquitin-binding scaffold protein that links LC3B to autophagic degradation. We observed a higher accumulation of LC3B-II and P62/SQSTM1 in GRN+/− fibroblasts when compared to CTL fibroblasts (*p* < 0.05) (Figure 3a, uncropped images in Figure S9).

To corroborate this finding, we performed a quantitative analysis of autophagosomes through LC3B immunofluorescence, a technique that is more sensitive than Western blot analysis, which revealed a significant increase in LC3B-II positive puncta within the GRN+/− fibroblasts compared to CTL fibroblasts (*p* < 0.05), under CM condition (Figure 3b). These findings suggest an increase in autophagosome formation in fibroblasts derived from FTD-GRN patients, which could be interpreted as a compensatory feedback mechanism for lysosomal dysfunction, similar to what has been proposed in LSDs [34]. Furthermore, analysis of TEM images revealed a striking accumulation of autophagosome structures, approximately threefold higher, in GRN+/− fibroblasts compared to CTL fibroblasts cultured under CM conditions (*p* < 0.05) (Figure 3c, detail in Figures S10 and S11). Taken together, these data allow us to conclude that autophagosomes accumulate in GRN+/− fibroblasts. In the context of the lysosomal dysfunction observed in these cells, one possible explanation is the activation of a compensatory response aimed at increasing autophagosome formation. However, as autophagic flux was not directly measured, this interpretation remains tentative, and alternative mechanisms (such as impaired autophagosome degradation) cannot be excluded. Nonetheless, *GRN*-mutant fibroblasts recapitulate key lysosome-related features of FTD and therefore provide a useful model for investigating lysosomal dysfunction and downstream lysosome-associated alterations relevant to the disease.

### 3.4. GRN Haploinsufficiency Causes Mitochondrial Abnormalities in Skin Fibroblasts from FTD-GRN Patients

**Figure 4:**
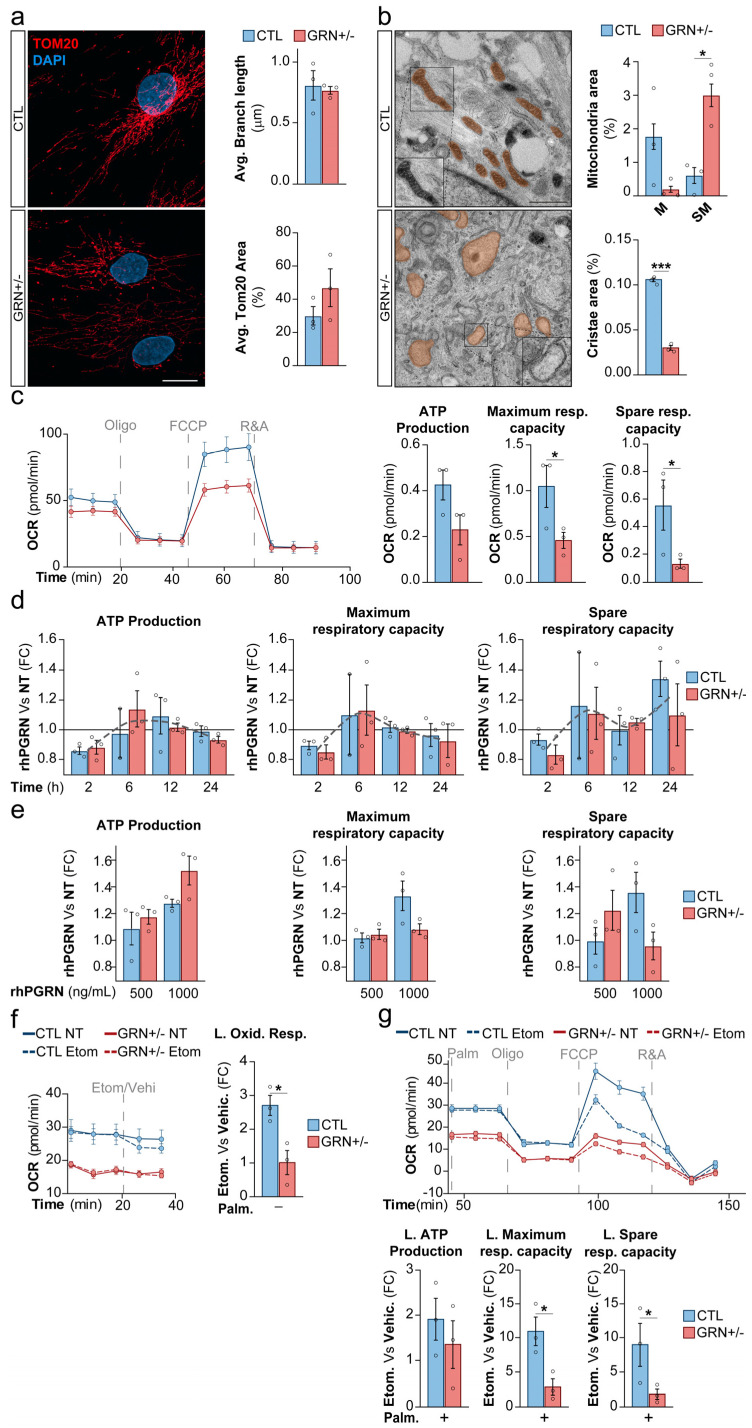
Altered Mitochondrial Function in Fibroblasts from Heterozygous *GRN*c.709-1G>A Mutation Carriers. (**a**) Representative images of TOM20 (red) and DAPI (blue) staining in primary human fibroblasts. Scale bar: 20 µm. (**b**) Representative TEM images of primary human fibroblasts, showing mitochondrial ultrastructure (orange). Insets: (top) Functional mitochondria, (bottom) Swollen mitochondria. Scale bar: 1 µm. (**c**) Seahorse assay depicting mitochondrial oxygen consumption in primary human fibroblasts. (**d**) Seahorse assay measuring mitochondrial oxygen consumption in primary human fibroblasts cultured with or without the specified pre-assay period and treated with rhPGRN (500 ng/mL) at different time points (2, 6, 12, and 24 h). (**e**) Seahorse assay analyzing mitochondrial oxygen consumption in primary human fibroblasts cultured with or without a 6 h pre-assay period and exposed to rhPGRN (500 ng/mL or 1000 ng/mL). (**f**) Seahorse assay assessing mitochondrial free fatty acid oxygen consumption in primary human fibroblasts. Left panel: Time-course evolution of the basal OCR. Right panel: Quantification of lipidic oxidative capacity, represented as the change in OCR induced by etomoxir treatment. (**g**) Seahorse assay measuring mitochondrial free fatty acid oxygen consumption in primary human fibroblasts. Top panel: Time-course evolution of the OCR during the assay. Bottom panel: Quantification of lipidic ATP production, maximal respiratory capacity, and spare respiratory capacity, represented as the change in these parameters induced by etomoxir treatment. Mitochondria (M) Swollen mitochondria (SM), Lipidic (L), Palmitic acid (Palm). Data are shown as mean ± SEM of independent cell lines (*n* = 3 per group) from one experiment. Each data point represents the mean of 1–8 technical replicates for a single line depending on the assay. The experiment was repeated independently three times with similar results. Statistical significance is indicated as follows: * *p* < 0.05 and *** *p* < 0.001.

To investigate whether *GRN* haploinsufficiency adversely affects cellular metabolism, given the critical interaction between mitochondria and lysosomes in metabolic processes, we analyzed the mitochondrial status in *GRN* haploinsufficient fibroblasts. Quantification of the mitochondrial outer membrane marker TOM20 by immunofluorescence revealed no discernible impact on either the mitochondrial surface area or mitochondrial network integrity, measured by mitochondrial branch length (Figure 4a). However, ultrastructural assessment using TEM revealed a pronounced escalation in the prevalence of swollen mitochondria (*p* < 0.05) and a concomitant reduction in the abundance of mitochondrial cristae within GRN+/− fibroblasts when compared to the CTL group (*p* < 0.005) (Figure 4b, detail in Figures S12 and S13). In *GRN* haploinsufficient fibroblasts, we notice a disruption specifically in the inner mitochondrial membrane (IMM), rather than the outer mitochondrial membrane (OMM). This parallels diseases resulting from IMM mutations, such as Leigh syndrome associated with alterations in *SLC25A46*, where IMM disturbances occur independently of OMM changes [35]. Mitochondrial cristae are essential cellular structures for energy production, containing the enzymes required for oxidative phosphorylation.

To investigate the functional capacity of these swollen mitochondria, we performed an assessment of mitochondrial respiration using the SeaHorse technology. Our findings indicate that the basal ATP production may be diminished in FTD-GRN fibroblasts, although this analysis did not reach statistical significance due to the notable variability of CTL fibroblasts (Figure 4c). However, a significant impairment in maximal respiration capacity and spare respiratory capacity was distinctly evident in GRN+/− fibroblasts (*p* < 0.05), attributed to a concurrent reduction in spare respiration when compared to CTL fibroblasts (*p*< 0.05) (Figure 4c). To elucidate the potential modulation of mitochondrial respiratory capacity by PGRN, we treated fibroblasts with 500 ng/mL of rhPGRN for varying durations. Our findings showed that rhPGRN treatment displayed the capacity to enhance mitochondrial ATP production and elevate maximal respiratory capacity and spare respiratory capacity, particularly in GRN+/− fibroblasts. Interestingly, this effect was not evident at the 2 h time point following treatment initiation, a period during which rhPGRN did exhibit functional activation of lysosomes (Figure 2e). Instead, improvements were seen at later time points, especially between 6 and 12 h (Figure 4d). In addition to these time-dependent effects, our study also found dose-dependent responses. Specifically, using rhPGRN at a concentration of 1000 ng/mL led to a noticeable increase in ATP production as early as 6 h after treatment (Figure 4e). However, this increase did not affect the maximal respiratory capacity or spare respiratory capacity (Figure 4e). These findings provide compelling evidence of marked mitochondrial abnormalities in GRN+/− fibroblasts, characterized by impaired respiratory capacity. While rhPGRN treatment partially ameliorates this defect, the effect size is modest. Notably, although basal ATP production appears sufficient, these mitochondria may be unable to meet increased energy demands under stress conditions.

After identifying alterations in mitochondrial respiration in patient-derived fibroblasts, we sought to determine whether the mitochondrial defects observed in GRN+/− fibroblasts were specific to a particular energy source. When both GRN+/− and CTL fibroblasts were subjected to a 6 h STV condition prior to SeaHorse analysis, a reduction in mitochondrial respiratory capacity was observed in both CTL and patient-derived cells (Figure S14a). Since nutrient deprivation is known to reduce mitochondrial respiration, and lysosomal dysfunction and autophagosme accumulation are also related to a STV metabolic phenotype [36], we sought to study the mitochondrial β-oxidation in PGRN-deficient fibroblasts.

To assess FAO, we inhibited mitochondrial fatty acid import using etomoxir, an irreversible inhibitor of carnitine O-palmitoyltransferase 1 (CPT1), and measured the oxygen consumption rate (OCR) using the Seahorse assay. OCR values obtained in the presence of etomoxir reflect non–fatty acid–FAs derived respiration, as FAs entry into mitochondria is blocked. In contrast, OCR measured under vehicle conditions represents total mitochondrial respiration. The difference between vehicle- and etomoxir-treated conditions was therefore used as an estimate of FAO-dependent respiration, with a larger difference indicating higher reliance on FAO. After the inhibition of FAO with Etomoxir, CTL fibroblasts exhibited a more pronounced reduction in OCR compared to patient-derived fibroblasts. This suggests that patient-derived fibroblasts are less dependent on FAs for oxidative respiration, as evidenced by the smaller difference in OCR between Etomoxir-treated and untreated cells (Figure 4f). To evaluate lipid-based ATP production, maximal lipid respiratory capacity, and lipid spare respiratory capacity, we measured the difference in OCR between Etomoxir-treated and untreated fibroblasts under conditions of palmitic acid surplus. This approach ensures that the availability of FAs does not mask the results, allowing us to accurately assess the capacity of the mitochondria to metabolize FAs. As it is shown in Figure 4g, lipidic ATP production may be diminished in GRN+/− fibroblasts, although this analysis did not reach statistical significance due to the notable variability of CTL fibroblasts. However, a significant impairment in Lipidic maximal respiration capacity and Lipidic spare respiratory capacity was distinctly evident in GRN+/− fibroblasts (*p* < 0.05), attributed to a concurrent reduction in spare respiration (*p* < 0.05), when compared to CTL fibroblasts (Figure 4g, detail in Figure S14b).

To confirm that FAs transport is intact and that the reduced effect of Etomoxir is not due to impaired FAs transport into the mitochondria, we tracked Red-C12, a saturated FAs analog with a fluorophore with an overall length approximately equivalent to that of an 18-carbon FA, in both CTL and GRN+/− fibroblasts. We observed a similar accumulation of Red-C12 within the mitochondria of GRN+/− fibroblasts, as indicated by Red-C12 staining in Mitotracker-positive structures (Figure S15). This suggests that FAs transport into the mitochondria is not compromised.

Our data indicate that GRN haploinsufficiency is associated with reduced mitochondrial FAO, as control cells exhibit higher FAO levels compared to GRN+/− fibroblasts. This reduction is accompanied by impaired mitochondrial respiratory performance, consistent with altered mitochondrial metabolism. This suggests dysfunctional mitochondrial respiration that may increase the likelihood of lipid accumulation.

### 3.5. GRN Insufficiency Causes Impaired Lipid Metabolism in Skin Fibroblasts from FTD-GRN Patients

**Figure 5:**
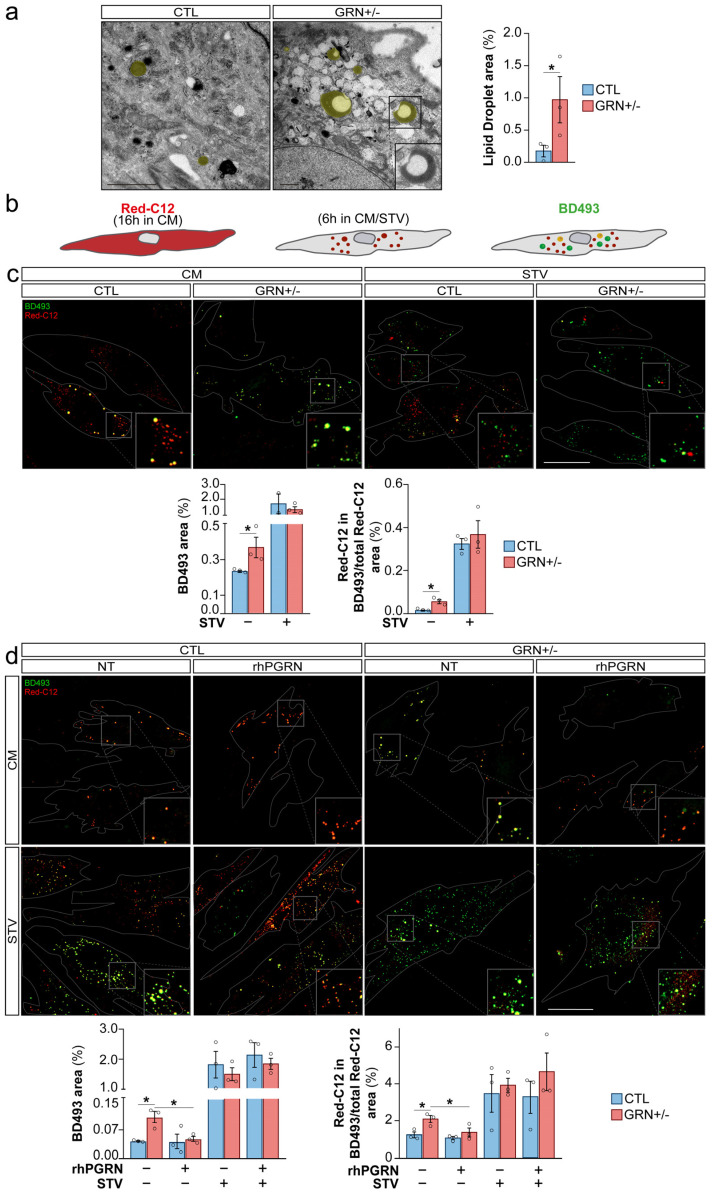
Disrupted Lipid Metabolism in Fibroblasts from Heterozygous *GRN*c.709-1G>A Mutation Carriers. (**a**) Representative TEM images of primary human fibroblasts, showing lipid droplets (LDs, yellow). Inset: High-magnification view of a LD exhibiting liquid–liquid phase separation of lipid components, characterized by an electron-lucent core and an electron-dense capsule. Note the direct interaction of the membrane-less LD surface with a network of vimentin intermediate filaments. Scale bar: 2 μm. (**b**) Schematic depiction of the pulse-chase assay. (**c**) Representative images of BODIPY 493/503 (green) and BODIPY 558/568 C12 (red) staining in primary human fibroblasts cultured under normal or starvation (STV) conditions (6 h). Bar plots: Quantification of the area covered by BD493-positive LDs and the proportion of Red-C12 staining within BD493-positive LDs. Scale bar: 50 µm. (**d**) Representative images of BD493 (green) staining in primary human fibroblasts cultured under normal or STV conditions and treated with rhPGRN (500 ng/mL) for 2 h. Bar plots: Quantification of the area covered by BD493-positive LDs and the proportion of Red-C12 staining within BD493-positive LDs. Scale bar: 50 µm. Data are shown as mean ± SEM of independent cell lines (*n* = 3 per group) from one experiment. Each data point represents the mean of 1–8 technical replicates for a single line depending on the assay. The experiment was repeated independently three times with similar results. Statistical significance is denoted as follows: * *p* < 0.05.

Accordingly, our focus shifted towards intracellular organelles termed LDs, which act as reservoirs for stored lipids destined for future utilization. Analysis of LDs by TEM images unveiled a significant rise in these structures in GRN+/− fibroblasts compared to CTL fibroblasts (*p* < 0.05), often located proximal to or in direct contact with the endoplasmic reticulum, and characterized by an electron-lucent core encompassed by an electron-dense periphery (Figure 5a, detail in Figures S16 and S17). This pattern is consistent in GRN+/− fibroblasts where the incorporation of newly synthesized TAGs which are more electron-dense, into pre-existing LDs enriched with cholesterol esters which are electron-lucent [37], results in liquid-to-liquid phase separation of these two lipid components.

This discovery prompted us to delve into how the regulation of LD dynamics is affected in GRN+/− fibroblasts and to elucidate whether treatment with rhPGRN can exert a modulatory influence on this process. To monitor the recent incorporation of esterified FAs into LDs, we implemented a pulse-chase assay using Red-C12 and BD493, which incorporates into LD-specific neutral lipids (Figure 5b) [23]. Under CM condition GRN+/− fibroblasts demonstrated elevated levels of BD493 staining in comparison to CTLs (*p* < 0.05), as indicated in Figure 5c (uncropped images in Figure S18). This observation is in accordance with our TEM imaging findings (Figure 5a) and is further supported by a larger portion of Red-C12 being localized within LDs in GRN+/− fibroblasts in comparison to CTLs (*p* < 0.05) (Figure 5c). The elevated LD levels observed in PGRN haploinsufficient fibroblasts suggest lipid dysfunction in these mutant cells.

The dynamics of LDs are tightly regulated, especially under conditions of nutrient deprivation. During such periods, FAs can be mobilized from membrane-bound organelles through autophagy and lysosomal lipolysis, thereby augmenting the LD pool. Conversely, FAs can be released from LDs themselves by lysosomal (lipophagy) and cytoplasmic lipolysis, facilitating their transfer into mitochondria [20,23].

As expected, our observations revealed an increase in LD formation under STV conditions, a phenomenon that was similar in both CTL and GRN+/− fibroblasts. This was evident through both increased BD493 staining of LDs and, importantly, the vast majority of Red-C12 being localized within LDs in GRN+/− fibroblasts compared to CTLs (*p* < 0.05) (Figure 5c). The observed reduction in differences under nutrient deprivation could be attributed to either a saturation point in the cells’ ability to store LDs, or a reduced biogenesis of LDs through autophagolysosome-driven processes in GRN+/− fibroblasts. However, considering that lipid dynamics shift towards LDs accumulation and increased FAs metabolism under nutrient scarcity, the accumulation of LDs observed under CM condition in GRN haploinsufficient fibroblasts resembles the response observed during nutrient deprivation [23]. This finding suggests that PGRN-deficient cells may exhibit features that more closely resemble a STV-like phenotype.

To determine whether the observed alterations in LD dynamics within GRN+/− fibroblasts were directly attributable to GRN insufficiency, we treated both CTL and GRN+/− fibroblasts with rhPGRN at 500 ng/mL for 2 h. As depicted in Figure 5d, the 2 h rhPGRN treatment in GRN+/− fibroblasts showed a significant reduction in LD accumulation when compared to GRN+/− NT fibroblasts (*p* < 0.05), effectively restoring up to CTL fibroblast levels. It is noteworthy that this effect was specific to GRN+/− fibroblasts, as rhPGRN treatment did not modify LD levels within CTL fibroblasts (Figure 5d, uncropped images in Figure S19). rhPGRN treatment in GRN+/− fibroblasts reduced the proportion of Red-C12 in LDs when compared to GRN+/− NT fibroblasts (*p* < 0.05), bringing levels to those observed in CTLs under CM conditions (Figure 5d). However, this treatment showed no effect under nutrient-deprived conditions (Figure 5d), likely because the strong impact of the STV condition masked the effect of rhPGRN. In any case, under CM conditions, LD dynamics appear to be directly influenced by PGRN leading to lipid dysfunction in *GRN* haploinsufficient fibroblasts.

## 4. Discussion

This study investigated the cellular mechanisms underlying FTD in *GRN* c.709-1G>A mutation carriers. *GRN* insufficiency was found to result in a complex interplay of cellular alterations in GRN+/− fibroblasts, providing new insights into the disease mechanisms.

Comparison of fibroblasts from FTD-GRN patients and healthy CTLs revealed several key pathological hallmarks, similar to those observed in the brains of FTD patients. We confirmed reduced GRN protein and mRNA levels in GRN+/− fibroblasts, consistent with the haploinsufficiency seen in *GRN*-associated FTD [29,30]. We also observed impaired lysosomal acidification and dysregulated autophagic flux, likely contributing to the accumulation of storage material. This phenomenon is well-documented in the cerebral cortex of FTD-GRN patients [33] and is consistent with reports of lysosomal abnormalities and the accumulation of storage material in various experimental models, including animal studies [38,39] and patient-derived extraneuronal cell cultures, such as fibroblasts [14]. Although fibroblasts are peripheral cells, they share key PGRN-related pathways with neurons, including expression of several membrane receptors and reliance on conserved endocytic and lysosomal mechanisms. In both cell types, PGRN deficiency leads to lysosomal dysfunction, a central feature of *GRN*-associated neurodegeneration, making fibroblasts a relevant system to study core cellular consequences of PGRN haploinsufficiency. At the same time, we acknowledge that fibroblasts do not fully capture the specialized features and functional demands of neurons. Taken together, these findings reinforce the relevance of patient-derived fibroblasts as a suitable initial model for investigating specific cellular mechanisms associated with *GRN* haploinsufficiency, while also highlighting the need for complementary studies in CNS-derived cells and in vivo models to fully address disease complexity.

Mitochondrial dysfunction is a hallmark of various neurodegenerative diseases, including FTD [40]. Although only a few studies have specifically linked mitochondrial alterations to FTD-GRN [41,42]. In this study, we observed mitochondrial swelling and impaired respiratory function in GRN+/− fibroblasts, in line with previous reports suggesting that PGRN contributes to mitochondrial homeostasis in podocytes by regulating mitochondrial biogenesis, via the PGRN–Sirt1–PGC-1α/FoxO1 signaling pathway, and mitophagy [43]. These findings suggest that affected cells may struggle to meet elevated energy demands during stress conditions requiring increased ATP production. Furthermore, impaired mitochondrial function could lead to the accumulation of dysfunctional mitochondria, lipids, and reactive oxygen species, thereby exacerbating cellular damage.

Reflecting broader metabolic dysregulation, PGRN-haploinsufficient fibroblasts also displayed increased accumulation of LDs, indicative of impaired lipid homeostasis. Abnormal LD levels are commonly observed in neuronal cells from patients with neurodegenerative disorders, including FTD [44,45]. Notably, brain lipid composition in both humans and mice with PGRN deficiency exhibits disease-specific alterations [46]. Although LDs are not inherently toxic, mounting evidence suggests they may exert detrimental effects over time [47]. In LSDs, secondary lipid-based storage products are frequently observed and are believed to contribute directly to disease pathogenesis [48,49]. Similarly, lipofuscin accumulation in the central nervous system, once seen as a passive aging marker, is now understood as an active mechanism to sequester misfolded proteins. However, its buildup may ultimately worsen neurodegeneration by promoting the spread of toxic protein species [15]. In addition, changes in membrane composition driven by lipid imbalances can compromise membrane integrity and disrupt cellular signaling. The interaction between lipids and reactive oxygen species can generate peroxidated lipids, which are cytotoxic and can trigger cell death [50]. Lipid signaling has also been implicated in the regulation of neuroinflammation [51]. Taken together, these findings suggest that PGRN-deficient cells exhibit profound disruptions in lipid metabolism, potentially leading to altered membrane composition and impaired cell signaling. Combined with the accumulation of dysfunctional mitochondria, these defects may synergistically impair energy homeostasis and exacerbate the accumulation and propagation of toxic lipid and protein species, ultimately contributing to disease progression.

The relationship between lysosomal, mitochondrial, and lipid dysfunction is complex and not fully understood. Consistent with the observations, rhPGRN treatment produced the most rapid and pronounced effects on lysosomal function, followed by significant effects on lipid metabolism and, ultimately, mitochondrial function. The temporal sequence of effects may suggest, but does not establish, a model in which lysosomal recovery precedes improvements in downstream metabolic parameters, while alternative causal relationships remain possible. It is important to consider that extracellular rhPGRN can act through at least two complementary mechanisms: receptor-mediated signaling at the cell surface and intracellular uptake followed by lysosomal processing. Fibroblasts express sortilin, and extracellular PGRN is known to bind sortilin and prosaposin, undergo rapid endocytosis, and traffic to lysosomes where it is processed into mature granulin peptides [52]. In parallel, extracellular PGRN can directly signal through membrane receptors such as EphA2, Notch, TLR9, and TNFR, modulating cellular responses independently of internalization [9]. Our data indicate a strong link between PGRN function, lysosomal activity, and lipid metabolism. Notably, the lipid droplet accumulation and mitochondrial impairment observed in GRN+/− cells closely resemble the phenotype of control cells under nutrient deprivation. This suggests that lysosomal failure in FTD-GRN may produce a phenotypic resemblance to a ‘starvation-like’ metabolic state—characterized by lipid mobilization and reduced respiratory capacity—even in the presence of nutrients. In this context, the connection between *GRN* haploinsufficiency and mitochondrial dysfunction appears to be part of a broader bioenergetic shift secondary to lysosomal insufficiency. Further experiments targeting canonical nutrient-sensing pathways, such as AMPK and mTORC1, will be needed to fully elucidate the mechanistic basis of this relationship. The time-dependent effects observed in our study may therefore reflect an interplay between extracellular signaling and intracellular processing that ultimately converges at the lysosome to restore cellular nutrient homeostasis. Lysosomal dysfunction has been closely associated with both mitochondrial and lipid abnormalities in various neurodegenerative diseases and multiple LSDs [17,53,54]. In particular, studies on a specific form of neuronal NCL6 have reinforced the connection between lysosomal failure and defects in these two organelles [49,55]. However, similar findings have not yet been reported in NCL11, which is caused by *GRN* mutations—likely due to the limited number of identified cases and the lack of in-depth studies [56]. Lysosomes regulate lipid transport and biogenesis both directly, through the processing of lipids via saposins [57], and indirectly, via mechanisms involving the mTORC1 [58]. They also influence mitochondrial quality control through mitophagy. However, in the context of marked LD accumulation, it is plausible that lipid imbalance may lead to membrane composition alterations, which could in turn impair or aggravate mitochondrial homeostasis [59]. Our data indicates a close association between PGRN function, lysosomal activity, and lipid metabolism, whereas the relationship between *GRN* haploinsufficiency and mitochondrial dysfunction appears less direct. While lysosomal alterations may represent an important contributing factor to the observed lipid and mitochondrial changes, we cannot exclude parallel, bidirectional, or alternative pathways linking these processes. Further mechanistic studies will be required to clarify the hierarchy and causal relationships among lysosomal, lipid, and mitochondrial dysfunctions.

Lysosomal dysfunction in GRN+/− fibroblasts is associated with disruptions in lipid and mitochondrial homeostasis, resembling, in part, the phenotype observed under control starvation conditions, such as lipid droplet accumulation and impaired mitochondrial membrane potential. These disturbances reflect key features of lysosomal storage and neurodegenerative diseases and may contribute to disease-relevant cellular stress [60,61]. While lysosomal impairment, mitochondrial dysfunction and LD accumulation could potentially influence processes related to cellular aging or senescence, we did not directly assess canonical senescence markers (e.g., SA-β-gal, p16, or p21), and therefore these aspects remain a hypothesis for future investigation. Exploring how lysosomal dysfunction intersects with lipid and mitochondrial pathways, as well as potential senescence-like responses, represents a particularly interesting avenue for further research.

In conclusion, this study highlights the multifaceted cellular mechanisms underlying FTD-GRN pathology. The observed lysosomal dysfunction, altered autophagy, mitochondrial abnormalities, and impairments in lipid metabolism collectively contribute to the disease’s complexity. The data indicates that mitochondrial swelling and LD accumulation may play a role in the pathology of FTD-GRN. Together with established evidence of lysosomal dysfunction and autophagy alterations, our observations suggest a shift in the metabolic phenotype associated with *GRN* haploinsufficiency. This work opens several avenues for future research into FTD-GRN pathology and paves the way for identifying novel biomarkers to assess therapeutic efficacy and develop potential treatments. However, acknowledging the limited sample size, further research is needed to fully elucidate FTD-GRN pathogenesis and advance effective therapies.

## 5. Conclusions

In addition to impairing lysosomal function, *GRN* haploinsufficiency disrupts cellular metabolism, affecting both mitochondrial and lipid homeostasis. These metabolic alterations resulting from PGRN deficiency provide valuable insights into the fundamental pathological processes driving disease progression. Furthermore, acknowledging the limited sample size, these findings are hypothesis-generating and highlight pathways that warrant further investigation in neuronal and in vivo models, potentially guiding future research into therapeutic strategies, biomarkers, or other disease-relevant interventions for FTD-GRN.

## Data Availability

The original contributions presented in this study are included in the article/Supplementary Materials. Further inquiries can be directed to the corresponding authors.

## Supplementary Materials

The following supporting information can be downloaded at: https://www.mdpi.com/article/10.3390/cells15030276/s1, Figure S1: Uncropped Westen blot Images of Figure 1a; Figure S2: TEM image of healthy control primary human fibroblast 01; Figure S3: TEM image of FTD-GRN patient primary human fibroblast 01; Figure S4: TEM image of FTD-GRN patient primary human fibroblast 02; Figure S5: Uncropped Westen blot Images of Figure 2b; Figure S6: Uncropped Immunofluorescence Images of Figure 2d; Figure S7: Uncropped Immunofluorescence Images of Figure 2e; Figure S8: Uncropped Westen blot Images of Figure 3a; Figure S9: Extension of Figure 3; Figure S10: TEM image of healthy control primary human fibroblast 02; Figure S11: TEM image of FTD-GRN patient primary human fibroblast 03; Figure S12: TEM image of healthy control primary human fibroblast 03; Figure S13: TEM image of FTD-GRN patient primary human fibroblast 04; Figure S14: Extension of Figure 4; Figure S15: Fatty Acid Localization Inside Mitochondria; Figure S16: TEM image of healthy control primary human fibroblast 04; Figure S17: TEM image of FTD-GRN patient primary human fibroblast 05; Figure S18: Uncropped Immunofluorescence Images of Figure 5c; Figure S19: Uncropped Immunofluorescence Images of Figure 5d; Supplementary Methods. References [26,27,28] are cited in the Supplementary Materials.

## Abbreviations

The following abbreviations are used in this manuscript:

BafA1	Bafilomycin
BD493	BODIPY 493/503
BSA	Bovine serum Albumin
C9orf72	C9orf72-SMCR8 complex subunit
CLQ	Chloroquine
CM	Cell medium
CPT1	Carnitine O-palmitoyltransferase 1
CTL	Control
FAO	Fatty acid oxidation
FAs	Fatty acids
fFTD	Familial frontotemporal dementia
FTD	Frontotemporal Dementia
FTD-GRN	Frontotemporal Dementia due to granulin mutation
FUS	FUS RNA binding protein
GAPDH	Glyceraldehyde-3-phosphate dehydrogenase
GRN	Granulin
GRN+/−	GRN c.709-1G>A mutation carriers
LAMP-1	Lysosome-associated membrane glycoprotein 1
LAMP-2	Lysosome-associated membrane glycoprotein 2
LC3B	Microtubule-associated protein 1 light chain 3 beta
LC3B-II	Lipidated microtubule-associated protein 1A/1B-light chain 3
LDs	Lipid Droplets
LSD	Lysosomal storage disorders
MAPT	Microtubule associated protein tau
mTORC1	Mammalian target of rapamycin complex 1
NCL	Neuronal ceroid lipofuscinosis
NT	Non-treated
OCR	Oxygen consumption rate
P62/SQSTM1	Sequestosome 1
PGRN	Progranulin
Red-C12	BODIPY 558/568 C12
rhPGRN	Recombinant human progranulin
STV	Starvation
TAG	Triacylglycerol
TEM	Transmission electron microscopy
TOM20	Mitochondrial import receptor subunit TOM20 homolog
VCP	Valosin containing protein
