# Supplementary material for: Mitochondria and Lipid Defects in Hereditary Progranulin-Related Frontotemporal Dementia
Source: Cells. 2026 Feb 1;15(3):276. doi: 10.3390/cells15030276 (PMC12897215; doi:10.3390/cells15030276)
Supplement: Supplementary file 1 [file cells-15-00276-s001.zip › MDPI_Cells_Supplementary_Methods.pdf]

Supplementary Methods

A full description of all experimental procedures and detailed protocols is provided below; the conceptual framework and overall experimental design are summarized in the Main Methods section.

Human samples

A total of six skin biopsies were analyzed: three from carriers of the c.709-1G>A mutation in the *GRN* gene and three from control individuals without the mutation and with no evidence of neurological degeneration (Table 1). Control subjects were healthy relatives of the patients. All patients were treated at Donostia University Hospital following established consensus criteria published elsewhere [26]. Skin biopsies and genetic analyses were performed after obtaining written informed consent, in accordance with the regulations of the Ethics Committee of the Government of Basque Country.

Table 1. Characteristics of individuals enrolled in this study.

Characteristic	CTL	GRN+/-
Number of subjects	3	3
Age at biopsy	63.0±13	67.9±6
Age at onset	-	63.0±5
Gender, female:male	2:1	0:3
Genotype	Negative	c.709-1G>A

Values are expressed as mean ± SD.

Cell culture conditions

Primary fibroblast cultures were established from skin biopsies of healthy donors, healthy control fibroblasts (CTL), and GRN c.709-1G>A mutation carriers (GRN+/-). Fibroblast cells were cultured in cell medium (CM), consisting of DMEM/high glucose (Gibco) supplemented with 10% fetal bovine serum (FBS) (Gibco), 1.2% GlutaMAX (Thermo Fisher Scientific), and 100 mg/mL penicillin/streptomycin (Gibco). Cells were incubated at 37°C in a humidified atmosphere with 5% CO_2.

To study nutrient deprivation conditions, we used starvation (STV) medium, consisting of EBSS with Mg^{2+}, Ca^{2+}, and Phenol Red (Gibco), supplemented with $FeCl_3 \cdot 6H_2O$ (0.0669 mg/L), D-glucose (4.5 g/L), pyruvate (1 mM) (Gibco), MEM Vitamin Solution (100X), and 100 mg/mL penicillin/streptomycin (Gibco). Cells were maintained in this medium for 6 hours.

Treatments: 30 μM chloroquine (CQ) (Sigma-Aldrich) for 5 hours. 100 nM bafilomycin A1 (BafA1) (Selleckchem) for 2 hours. Recombinant human progranulin (rhPGRN) (R&D) at 500 ng/mL or 1000 ng/mL for 2, 6, 12, or 24 hours.

mRNA expression/processing analysis

Total RNA was extracted using the RNeasy Mini Kit with an RNase-free DNase Kit (Qiagen). Reverse transcription was performed using the SuperScript VILO cDNA Synthesis Kit (Thermo Fisher) following the manufacturer's instructions.

Quantitative real-time PCR (qRT-PCR) was conducted on a CFX384 Touch Real-Time PCR Detection System (Bio-Rad) using Power SYBR Green Master Mix (Thermo Fisher), 300 nM of each primer pair, and 10 ng of cDNA. Glyceraldehyde-3-phosphate dehydrogenase (*GAPDH)* was used as a housekeeping gene. Relative quantification was performed using the $2^{-\Delta\Delta Ct}$ method. Primer sequences: *GRN* (Forward: 5′-CTCTCCAAGGAGAACGCTACCA-3′; Reverse: 5′-GACTGTAGACGGCAGCAGGTAT-3′), *LAMP2* (Forward: 5′-TGACGACAACTTCCTTGTGC-3′; Reverse: 5′-AGCATGATGGTGCTTCAGAC-3′), *GAPDH* (Forward: 5′-ACAGTTGCCATGTAGACC-3′; Reverse: 5′-TTGAGCACGGGTACTTTA-3′).

Protein extraction and western blot

Primary fibroblasts were lysed in Cell Lysis Buffer (#9803, Cell Signaling) containing phosphatase and protease inhibitors (Thermo Fisher Scientific). Homogenates were centrifuged at 13,000× g for 15 minutes at 4°C.

Protein concentration was measured using a Pierce™ BCA Protein Assay Kit (#10741395, Thermo Fisher Scientific). Samples (10–20 μg) were resolved on loading buffer (0.16 M Tris-HCl, pH 6.8, 4% SDS, 20% glycerol, 0.01% bromophenol blue, 0.1 M DTT) and run on Mini-PROTEAN® TGX™ precast protein gels, Criterion™ TGX™ precast protein gels (Bio-Rad), or Tris-HCl/Tris-tricine gels. Proteins were transferred to Amersham™ Protran® nitrocellulose membranes (pore size 0.2 μm, GE Healthcare) overnight at 4°C at 15 V.

After blocking for 1 hour in 5% bovine serum albumin (BSA) in TBS-Tween, the membrane was incubated with specific primary antibodies overnight at 4°C.

Primary Antibodies: PGRN (1 μg/mL, AF2420, R&D Systems), LAMP-1 (1:10,000, ab25630, Abcam), LAMP-2 (1:1,000, #49067, Cell Signaling), LC3B (1:1,000, #3868, Cell Signaling), p62/SQSTM1 (1:1,000, #5114, Cell Signaling), β-Tubulin (1:5,000, MA5-16308, Thermo Fisher), TOM20 (1:1,000, 11802-1-AP, Proteintech).

Secondary antibodies included HRP-conjugated anti-mouse or anti-rabbit IgG (1:5,000, #7076, #7074, Cell Signaling) or fluorescent secondary antibodies, both incubated at 1:5,000 for 2 hours at room temperature (RT).

Protein bands were visualized using the iBright FL1000 Imaging System (Thermo Fisher) and quantified using Image Studio Lite software (LI-COR Biosciences). Immunoreactivity was calculated as the fold increase over the signal obtained in CTL samples. Protein levels were normalized to the β-Tubulin loading control.

Immunofluorescence

Primary fibroblasts were cultured in Ibidi μ-Slides or multi-well plates (μ-Plate 96 Well Black, #89626, Ibidi). Once an appropriate confluence was reached, cells were fixed in 4% paraformaldehyde for 20 minutes, followed by blocking and permeabilization with 5% BSA and 0.1% Triton X-100 in PBS for 1 hour at RT.

The primary antibodies used were anti-LC3B (#2775, Cell Signaling), LAMP-1 (ab25630, Abcam), and TOM20 (11802-1-AP, Proteintech), which were incubated overnight at 4°C. After three washes, cells were incubated with Alexa Fluor 488 or Alexa Fluor 647 secondary antibodies (Life Technologies) for 2 hours at RT, and chromatin was stained with DAPI (1 μg/mL, D1306, Thermo Scientific). A negative control was included by omitting the primary antibody. Samples were mounted using Ibidi mounting medium, and images were captured with a Zeiss LSM 900 confocal microscope, equipped with a live-cell imaging system with temperature and atmosphere control. Image analysis was performed using ImageJ, including colocalization analysis.

Fluorescence Quantification:

LC3 puncta and LAMP-2 intensity: The mean fluorescence intensity in the LC3 puncta or LAMP-2 channel was calculated across the entire image. The fluorescence intensity was normalized to the image area to determine the % optical density.

LC3 in mitochondria: A mask was created using the LC3 puncta channel, then overlapped with the TOM20 channel mask. Mitochondrial LC3 particle area was quantified using the "Analyze Particles" function in ImageJ, applied to thresholded images with size settings ranging from 0.1 to 100 μm^2 and circularity from 0 to 1. To determine LC3 % area per cell, the LC3 particles in the mitochondrial area were normalized to the total cell area, calculated using the Cellpose generalist algorithm for cell segmentation [27].

Mitochondrial area measurement: The "Analyze Particles" function in Image J (NIH) was used to quantify the mitochondrial area, applying a minimum threshold of 0.25 mm^2.

Mitochondrial network analysis: The “Skeletonize 2D/3D” command was applied to thresholded images, and the “Analyze Skeleton” function in Image J (NIH) was used to calculate the number of branches, branch length, and branch junctions in the skeletonized mitochondrial network.

All analyses were performed on whole cells.

Live Cell Imaging

Lysosomal Functionality Assay:

Fibroblast cells were incubated in CM containing 100 nM LysoTracker™ Red DND-99 (Thermo Fisher) for 5 hours. After incubation, cells were washed three times with CM and immediately imaged.

Fatty Acid Pulse and Chase Assay:

Fibroblasts were incubated in CM supplemented with 1 mM BODIPY 558/568 C12 (Red-C12, Life Technologies) for 16 hours. After incubation, cells were washed three times with CM, followed by a 1-hour incubation to allow fluorescent lipids to incorporate into LDs. Cells were then chased for the indicated duration in either CM or STV medium, in the absence or presence of rhPGRN. LDs were labeled with 200 ng/mL BODIPY 493/503 (BD493, Life Technologies) immediately prior to imaging, and this labeling was maintained throughout imaging.

Fatty Acid Tracking into Mitochondria:

Fibroblasts were incubated in CM containing 1 mM BODIPY 558/568 C12 (Red-C12, Life Technologies) for 16 hours. Cells were then washed three times with CM, followed by a 1 hour incubation to allow incorporation of fluorescent lipids. Cells were then chased for the indicated experimental timing. Mitochondria were labeled with 100 nM MitoTracker™ Green FM (M7514, Invitrogen™) for 30 minutes, followed by a single wash with CM before immediate imaging.

All images were acquired using a Zeiss LSM 900 confocal microscope, equipped with a time-lapse acquisition system and a live-cell imaging system with temperature and atmosphere control.

Image analysis was performed using ZEN Blue imaging software (Zeiss) and Image J (NIH). Brightness and contrast adjustments were made in Image J (NIH) as needed.

For Red-C12 fluorescence intensity in LDs, a mask was generated using the BD493 channel, which was then overlaid with the Red-C12 channel mask. The area of Red-C12 positive particles in LDs was quantified using the ImageJ “Analyze Particles” function in thresholded images, with size (μm^2) parameters set from 0.1 to 100 and circularity from 0 to 1. The percentage of Red-C12 area

per cell was determined by normalizing the Red-C12 particles within the LD area to the total cell area, which was calculated using the Cellpose generalist algorithm for cell segmentation.

LD (BODIPY 493/503) and lysosome (LysoTracker™ Red DND-99) particle areas were quantified using the ImageJ "Analyze Particles" function in thresholded images, applying the same size (μm^2) and circularity settings as described above. To calculate the percentage of LD or lysosomal area per cell, the LD or lysosomal particles were normalized to the total cell area, which was automatically generated using the Cellpose generalist cell segmentation algorithm.

Seahorse XF-96 Metabolic Flux Analysis

Oxygen consumption rates (OCRs) was measured using the Seahorse Bioanalyzer XF-96 (Agilent Technologies) according to the manufacturer's instructions. To standardize experimental data based on cell number, adherent cells in Seahorse XF-96 plates were stained with crystal violet, which binds to nucleic acids and proteins.

XF Cell Mito Stress Test

Cell Preparation: Primary fibroblasts were seeded at 18,000 cells per well in XF-96 plates one day before the assay. For STV conditions, cells were switched to STV medium for 6 hours prior to analysis.

Mito Assay Procedure: The assay was performed following the manufacturer's protocol. One hour before analysis, the culture medium was replaced with 175 µL of Mito assay medium, and cells were incubated at 37°C without CO_2 for 1 hour.

Mito Assay Medium Composition: 8.7 g/L MEM (61100-087, Thermo Fisher Scientific); 1 mM pyruvate (Gibco); 2 mM glutamine; 10 mM glucose; pH = 7.4.

Inhibitor Injections (sequentially added during the assay): Oligomycin 2 µM (ATP synthase inhibitor, Complex V); FCCP 2 µM (uncoupling agent); Rotenone 0.5 µM (Complex I inhibitor) and Antimycin A 0.5 µM (Complex III inhibitor).

OCR values were automatically calculated using the Seahorse XF-96 analyzer (Agilent Technologies).

Metabolic Parameters Calculations:

ATP Production = (Last OCR before Oligomycin injection) – (Minimum OCR after Oligomycin injection)

Maximal Respiratory Capacity = (Maximum OCR after FCCP injection) - (Minimum OCR after Rotenone/Antimycin A injection)

Spare Respiratory Capacity = (Maximal Respiratory Capacity) – (Basal Respiration)

Basal Respiration = (Last OCR before first injection) – (Minimum OCR after Rotenone/Antimycin A injection)

Fatty Acid Oxidation (FAO) Respiration Test

To assess FAO dependency, the Mito Stress Test Kit (Agilent Technologies) was used. This assay determines the reliance of fibroblasts on FAO for energy production, assessing whether cells can compensate for FAO inhibition by utilizing glucose or glutamine oxidation.

Cell Preparation: 48 hours prior to analysis: Fibroblasts were seeded at 18,000 cells/well in XF-96 plates. 24 hours before measurement medium was switched to substrate-limited medium to restrict metabolic flexibility. Immediately before the assay, the medium was replaced with FAO assay buffer, and cells were incubated at 37°C in a non-CO_2 incubator for 1 hour. Cells were then treated with 200 μM palmitate-BSA (29558, Cayman chem) or 34 μM BSA (29556 , Cayman Chem). Sequential Inhibitor Injections: Etomoxir 40 μM (FAO inhibitor); Oligomycin 2 μM; FCCP 2 μM; Antimycin A 0.5 μM → and Rotenone 0.5 μM.

FAO dependence calculations:

Lipidic Oxidative Response = (Etomoxir effect) – (Vehicle effect)

Vehicle Effect = (Last OCR before vehicle injection) – (Second OCR after vehicle injection)

Etomoxir Effect = (Last OCR before Etomoxir injection) – (Second OCR after Etomoxir injection)

Lipidic ATP Production = (ATP Production with vehicle) – (ATP Production with Etomoxir)

Lipidic Maximal Respiration = (Maximal Respiratory Capacity with vehicle) – (Maximal Respiratory Capacity with Etomoxir)

Lipidic Spare Respiration = (Spare Respiratory Capacity with vehicle) – (Spare Respiratory Capacity with Etomoxir)

Media and Buffer Preparation: Substrate-Limited Medium Composition: Seahorse XF DMEM (103575-100, Agilent); 0.5 mM D-Glucose (141341, ITW Reagents); 1 mM GlutaMAX™ (35050038, Gibco); 0.5 mM L-Carnitine (C0158, Sigma Aldrich); 1% FBS (10270-106, Gibco); pH = 7.4.

FAO Assay Buffer Composition: Seahorse XF DMEM (103575-100, Agilent); 0.5 mM L-Carnitine (C0158, Sigma Aldrich); pH = 7.4.

Transmission Electron Microscopy

Primary fibroblasts were fixed in 3% (v/v) glutaraldehyde in 0.1 M sodium phosphate buffer (pH 7.4) for 10 minutes at 37°C, followed by 2 hours at RT. Samples were then washed five times in 0.1 M sodium phosphate buffer (pH 7.4). The fixed cells were sent to the Electron Microscopy Core Facility at the Principe Felipe Research Center for further processing.

Ultrathin sections (70 nm) were imaged at the Core Facility for Polymer Characterization (UPV/EHU). The quantification of lysosomes, autophagosomes, fingerprint structures, lipid droplets (LDs), and mitochondrial area was performed in 10–15 cells per sample, with approximately 30–40 images acquired per clone, and the data were expressed as a percentage of the total cellular area (relative distribution). Cristae measurements were analyzed as described by Lam et al. [28].

Statistical Methods

Statistical analyses were performed using GraphPad Prism 8.0.0 software. All experiments were conducted in biological replicates, and data are presented as mean ± SEM from three independent experiments, unless otherwise stated.

Statistical significance was determined using the Mann-Whitney U test for comparisons between two independent groups. For comparisons involving multiple factors (e.g., GRN genotype × nutritional conditions, GRN genotype × PGRN treatment, PGRN treatment × time, or PGRN treatment × nutritional conditions), a two-way ANOVA followed by Tukey's post hoc test was applied.

Statistical significance in all figures is indicated as follows: non-significant (ns), $p > 0.05$, *$p < 0.05$, **$p < 0.01$, ***$p < 0.001$, ****$p < 0.0001$.
