# Supplementary material for: Mitochondria and Lipid Defects in Hereditary Progranulin-Related Frontotemporal Dementia
Source: Cells. 2026 Feb 1;15(3):276. doi: 10.3390/cells15030276 (PMC12897215; doi:10.3390/cells15030276)
Supplement: Supplementary file 1 [file cells-15-00276-s001.zip › Supplementary material_20260115.pdf]

Supplementary Materials for

Mitochondria and Lipid Defects in Progranulin-Related Frontotemporal Dementia.

Jon Ondaro *et al*.

*Corresponding authors: gorka.gerenu@biodonostia.org, francisco.gilbea@biodonostia.org

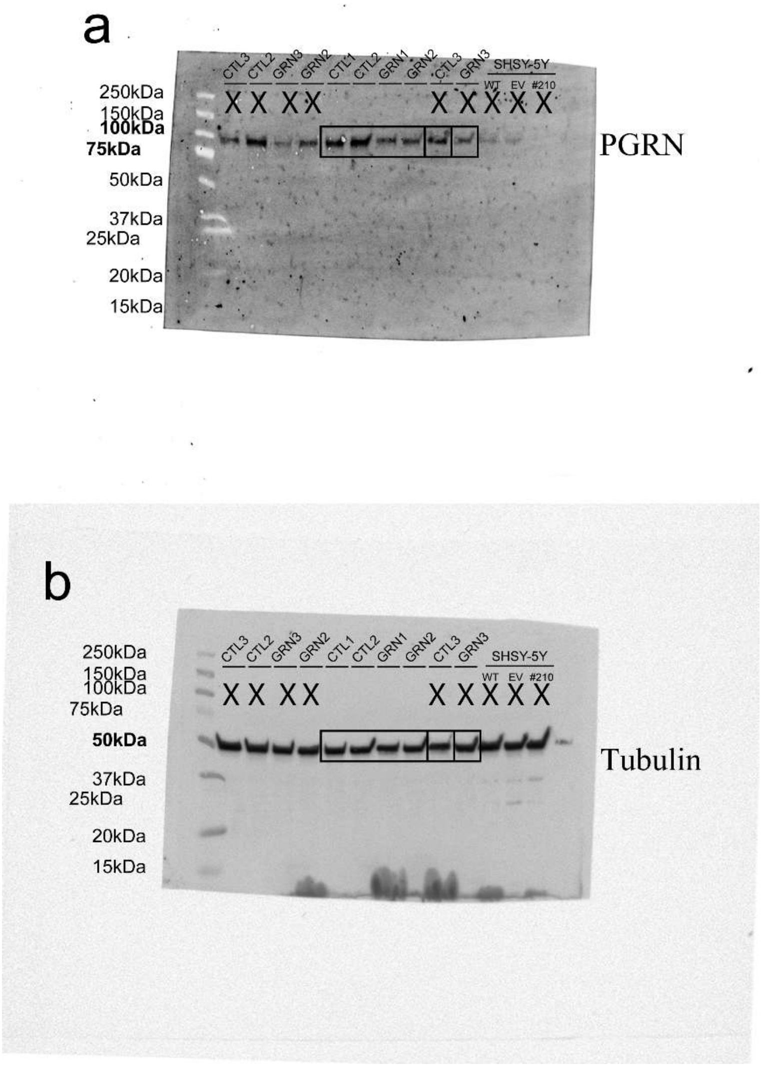

Figure S1. Uncropped Westen blot Images of Figure 1a. **a.** PGRN. **b.** Tubulin. Precision Plus Protein Dual Xtra Prestained Protein Standard (Bio-Rad, #1610377).

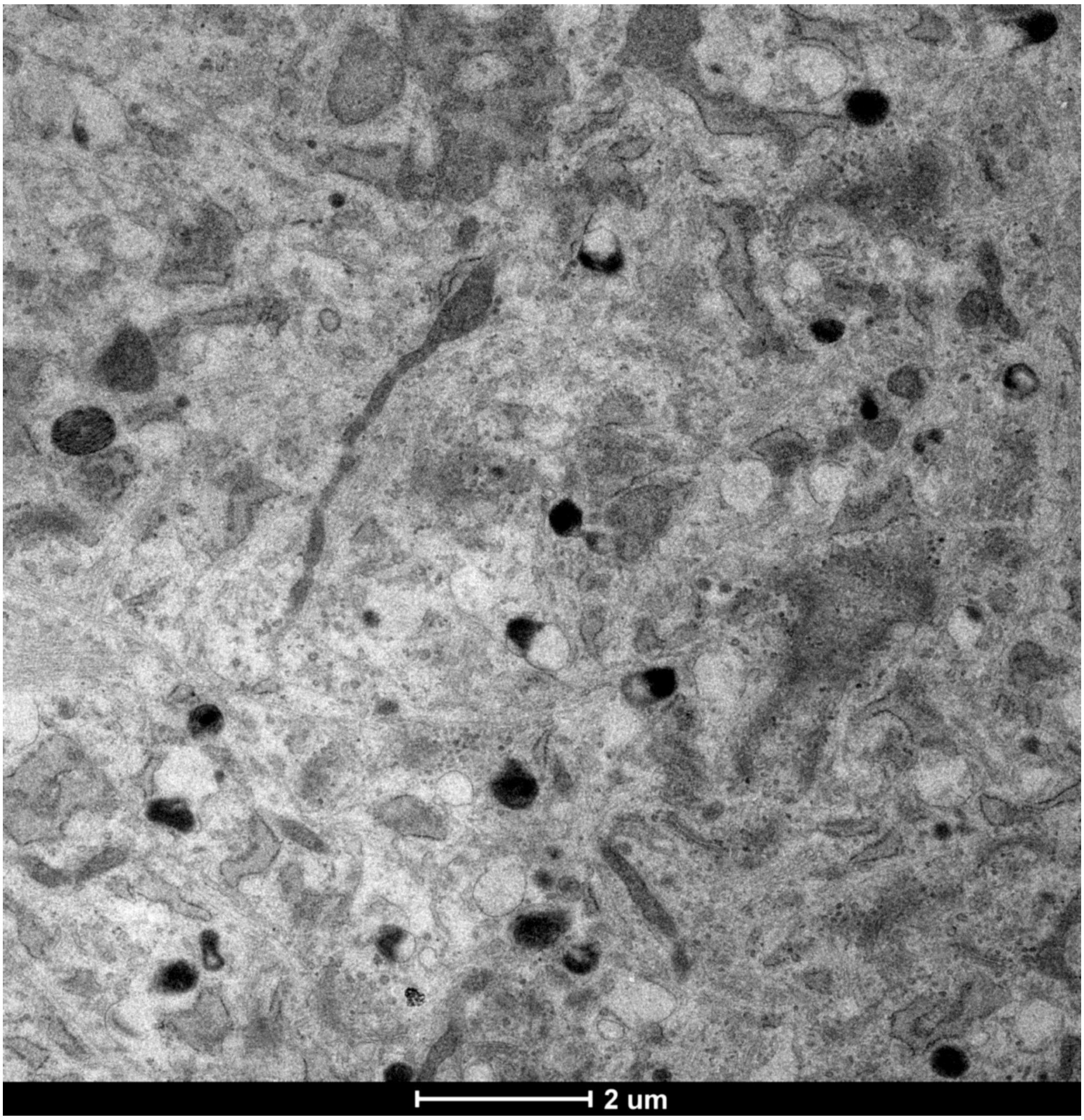

Figure S2. TEM image of healthy control primary human fibroblast 01. Scale bar: 2 μm.

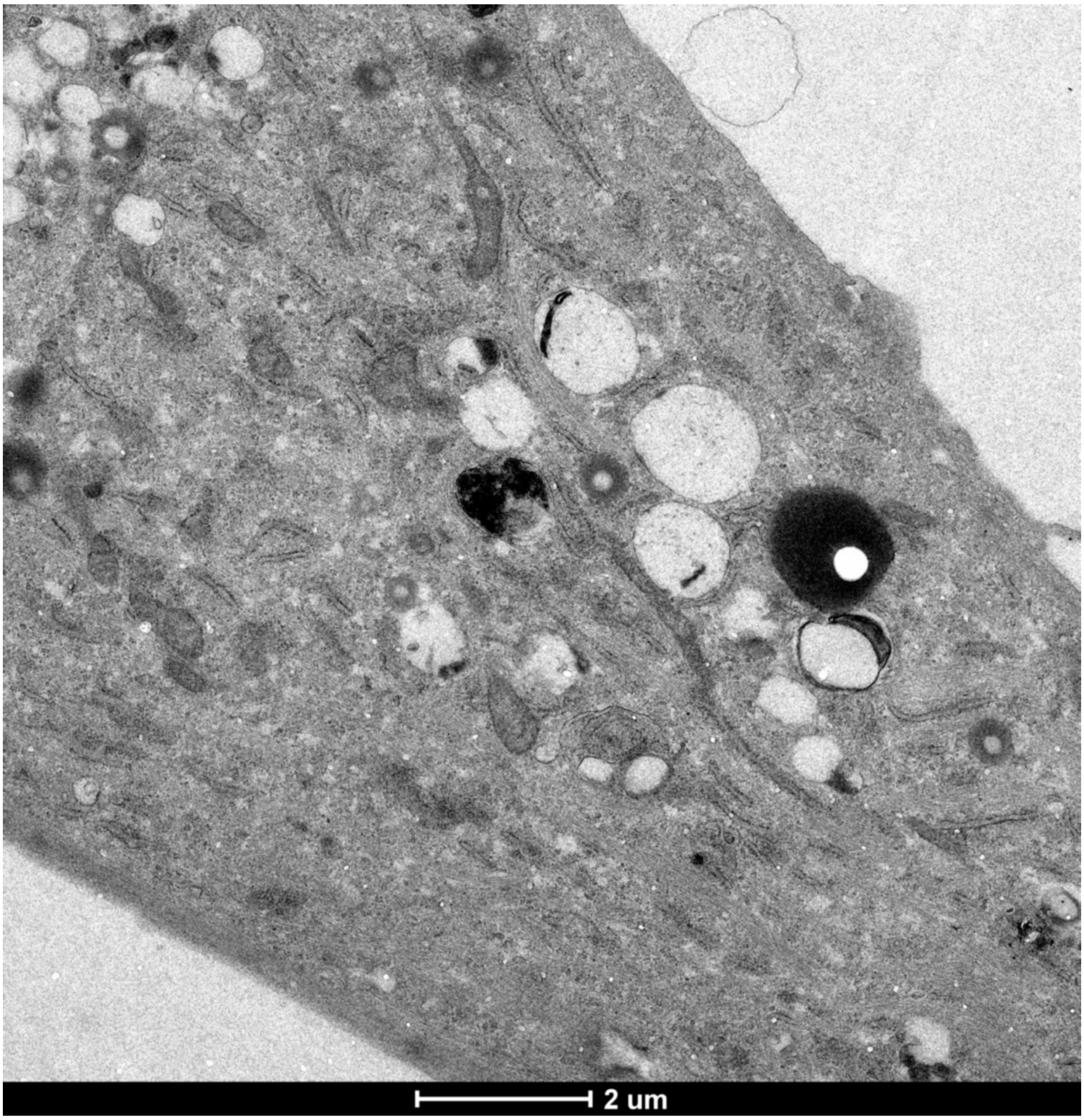

Figure S3. TEM image of FTD-GRN patient primary human fibroblast 01. Scale bar: 2 μm.

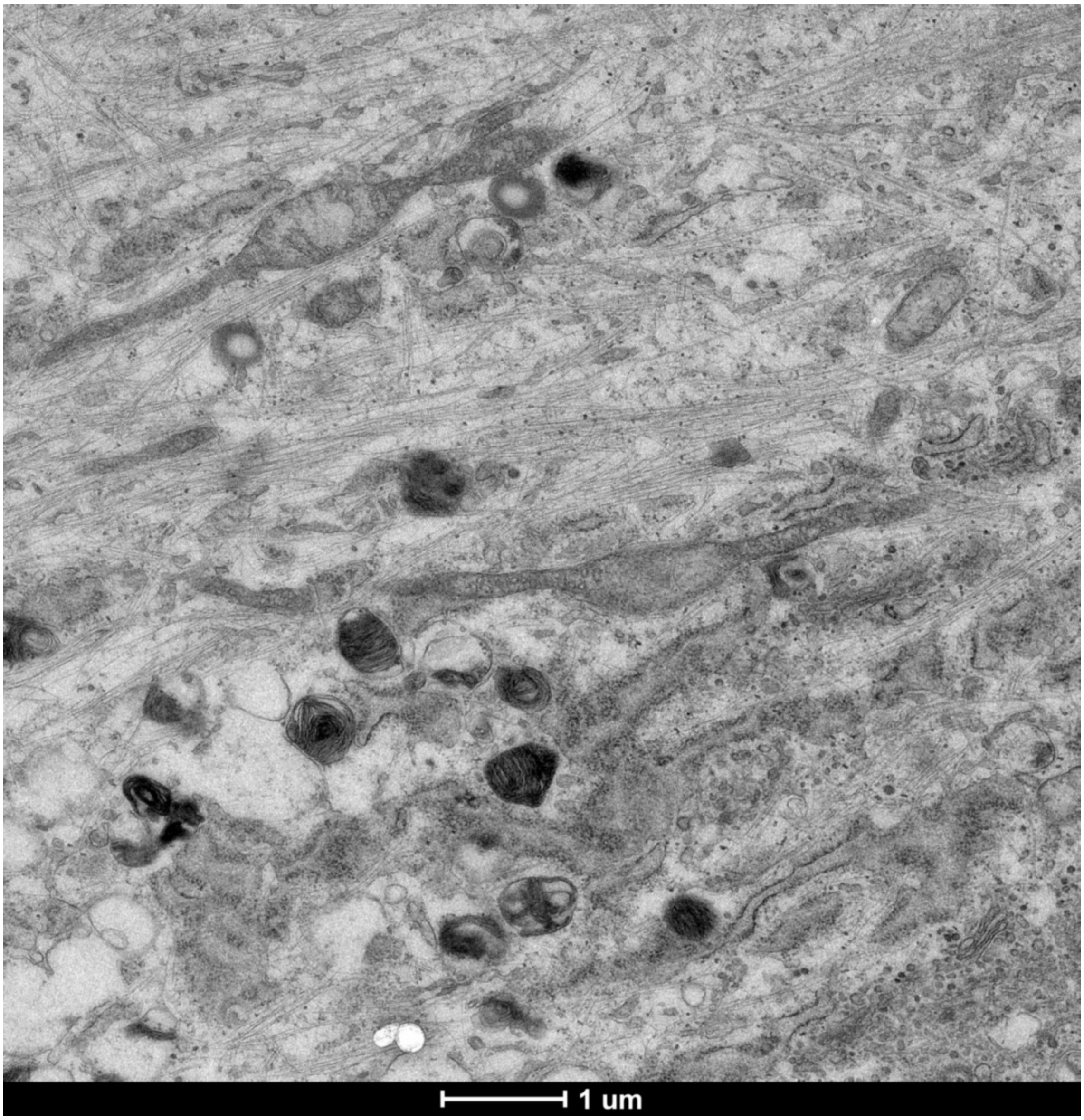

Figure S4. TEM image of FTD-GRN patient primary human fibroblast 02. Scale bar: 1 μm.

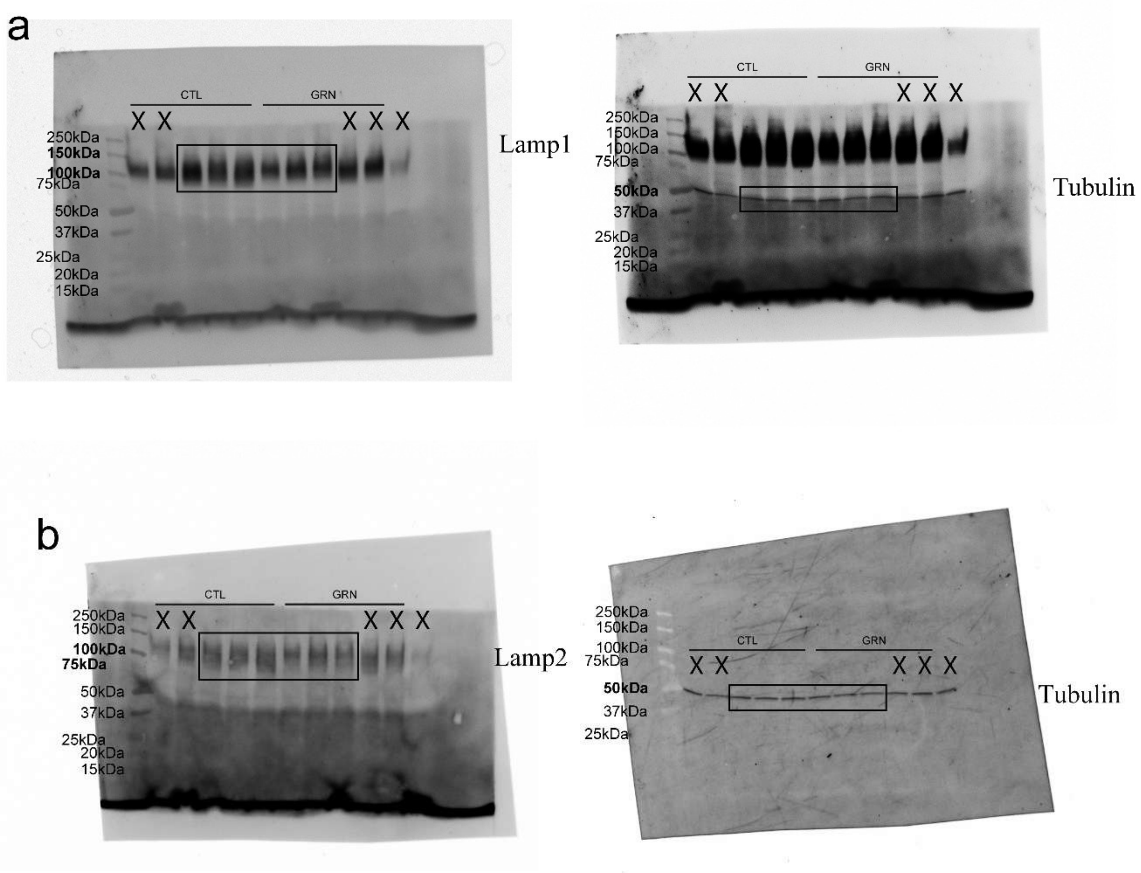

Figure S5. Uncropped Westen blot Images of Figure 2b. **a.** Membrane 1, Lamp1 and Tubulin. **b.** Membrane 2, Lamp2 and Tubulin. Precision Plus Protein Dual Xtra Prestained Protein Standard (Bio-Rad, #1610377).

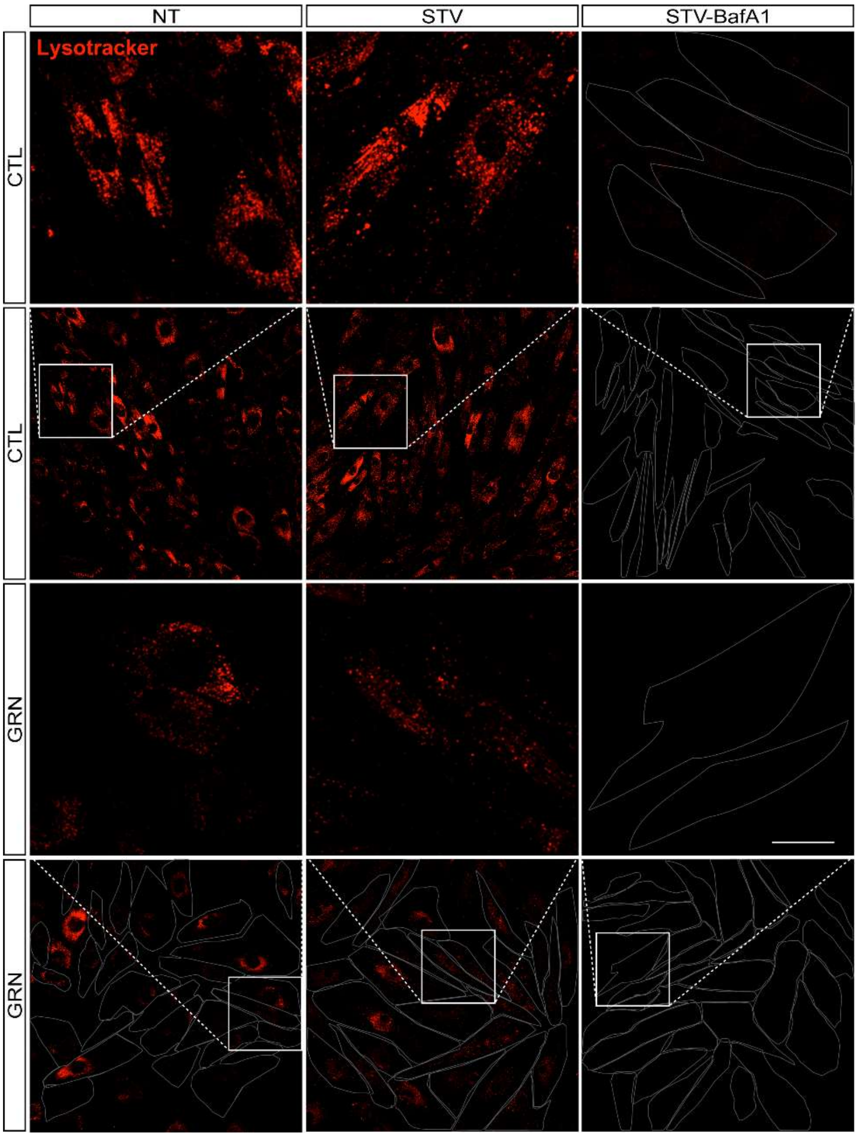

Figure S6. Uncropped Immunofluorescence Images of Figure 2d.

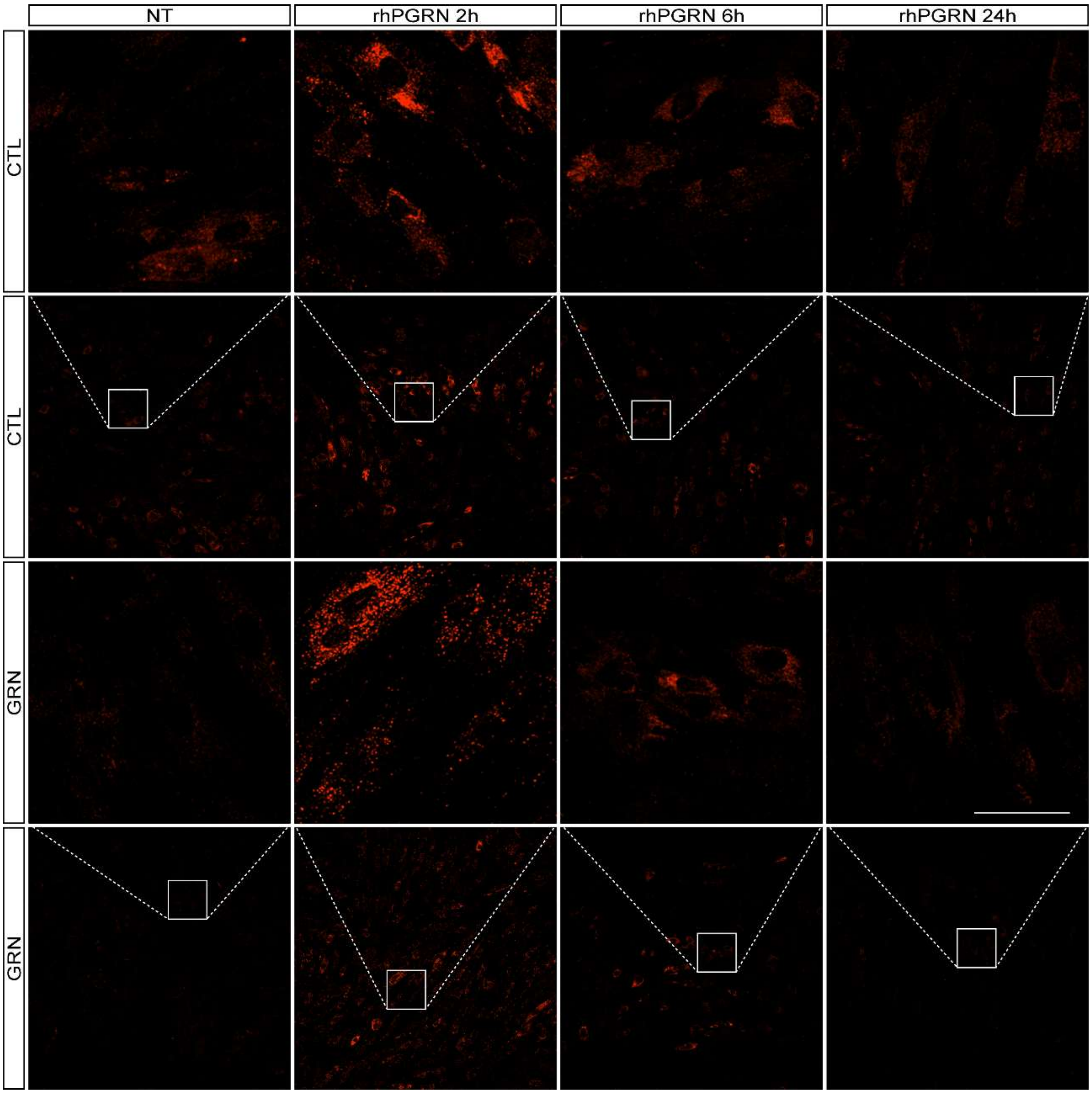

Figure S7. Uncropped Immunofluorescence Images of Figure 2e.

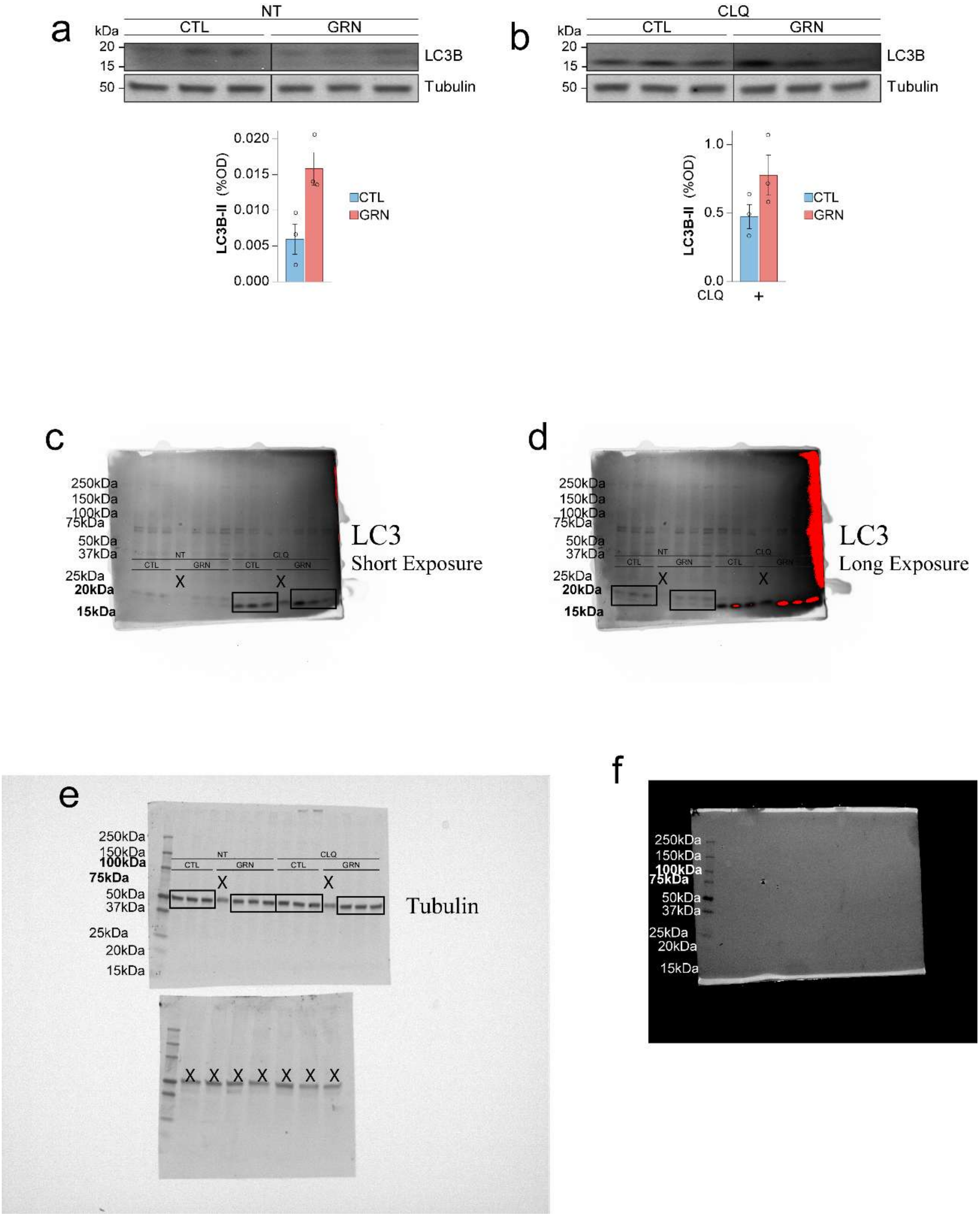

Figure S8. Uncropped Westen blot Images of Figure 3a. **a.** LC3B-II. **b.** SQSTM1/P62. **c.** Tubulin. Precision Plus Protein Dual Xtra Prestained Protein Standard (Bio-Rad, #1610377).

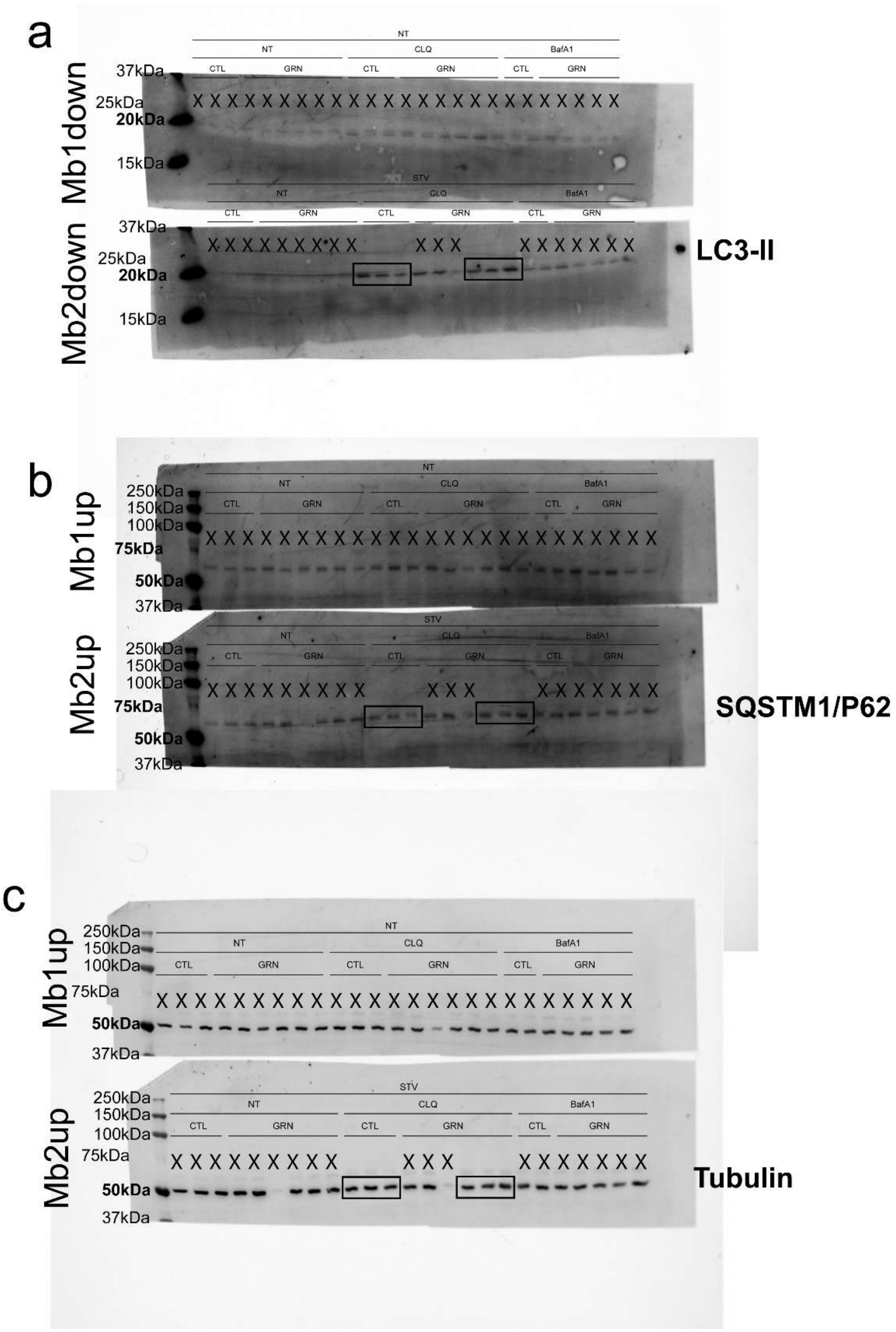

Figure S9. Extension of Figure 3. **a.** Protein levels of LC3B-II assessed by Western blotting in primary human fibroblasts. Western blot analysis was performed to assess LC3B-II protein levels, with quantifications shown as bar plots. **b.** Protein levels of LC3B-II assessed by Western blotting in primary human fibroblasts treated with chloroquine (CLQ, 30 µM for 5 h). Western blot analysis was performed to assess LC3B-II protein levels, with quantifications shown as bar plots. Healthy control fibroblasts (CTL), FTD-GRN patient fibroblasts (GRN), non-treated (NT), chloroquine (CLQ). Data are presented as mean ± SEM (n = 3). Statistical significance: $p > 0.05$, determined using Student's t-test. **c.** Uncropped Western blot Images of LC3, short exposure. **d.** Uncropped Western blot Images of LC3, Long exposure. **e.** Uncropped Western blot Images of Tubulin. **f.** Bright field image of Precision Plus Protein Dual Xtra Prestained Protein Standard (Bio-Rad, #1610377).

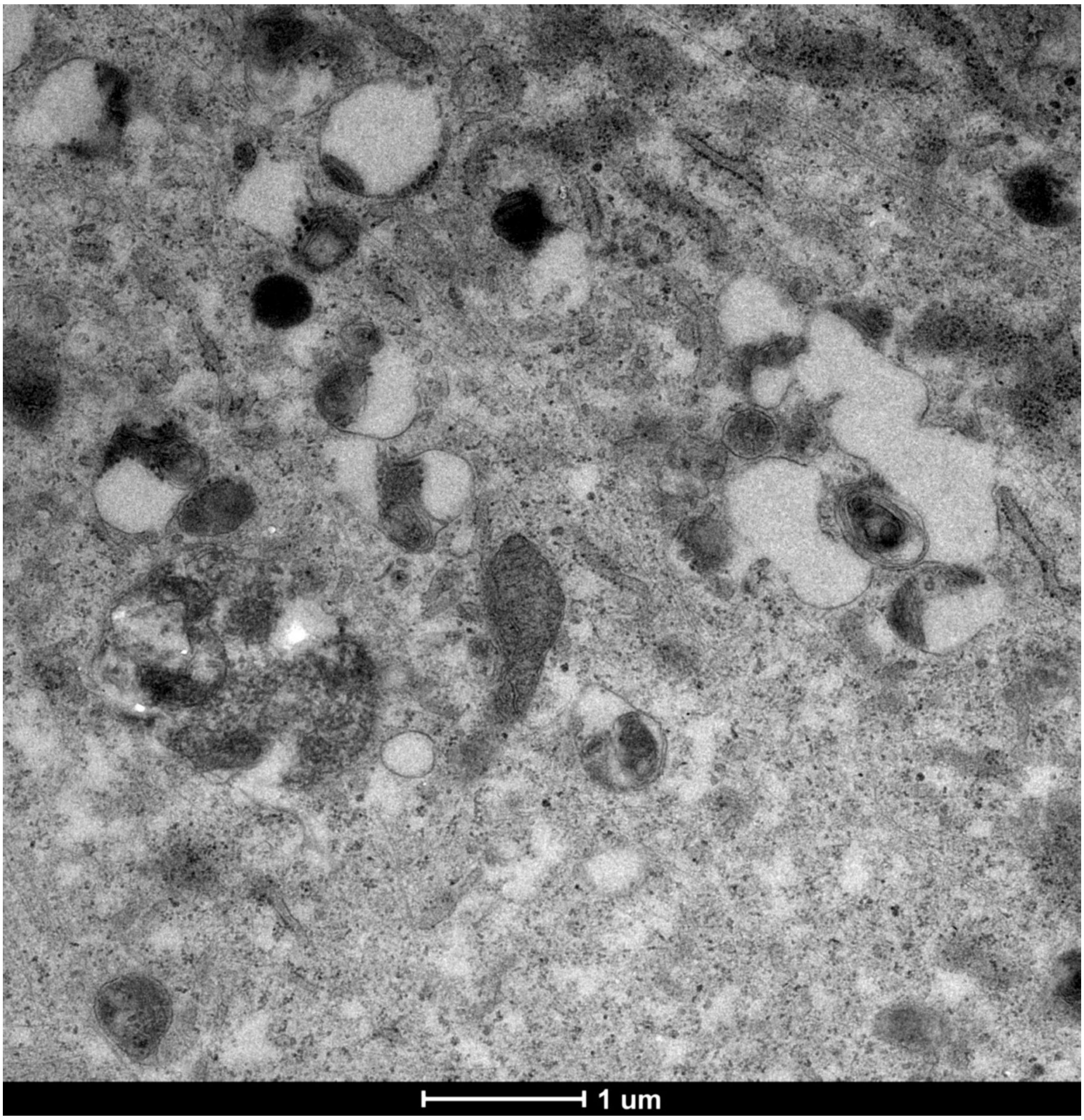

Figure S10. TEM image of healthy control primary human fibroblast 02. Scale bar: 1 μm.

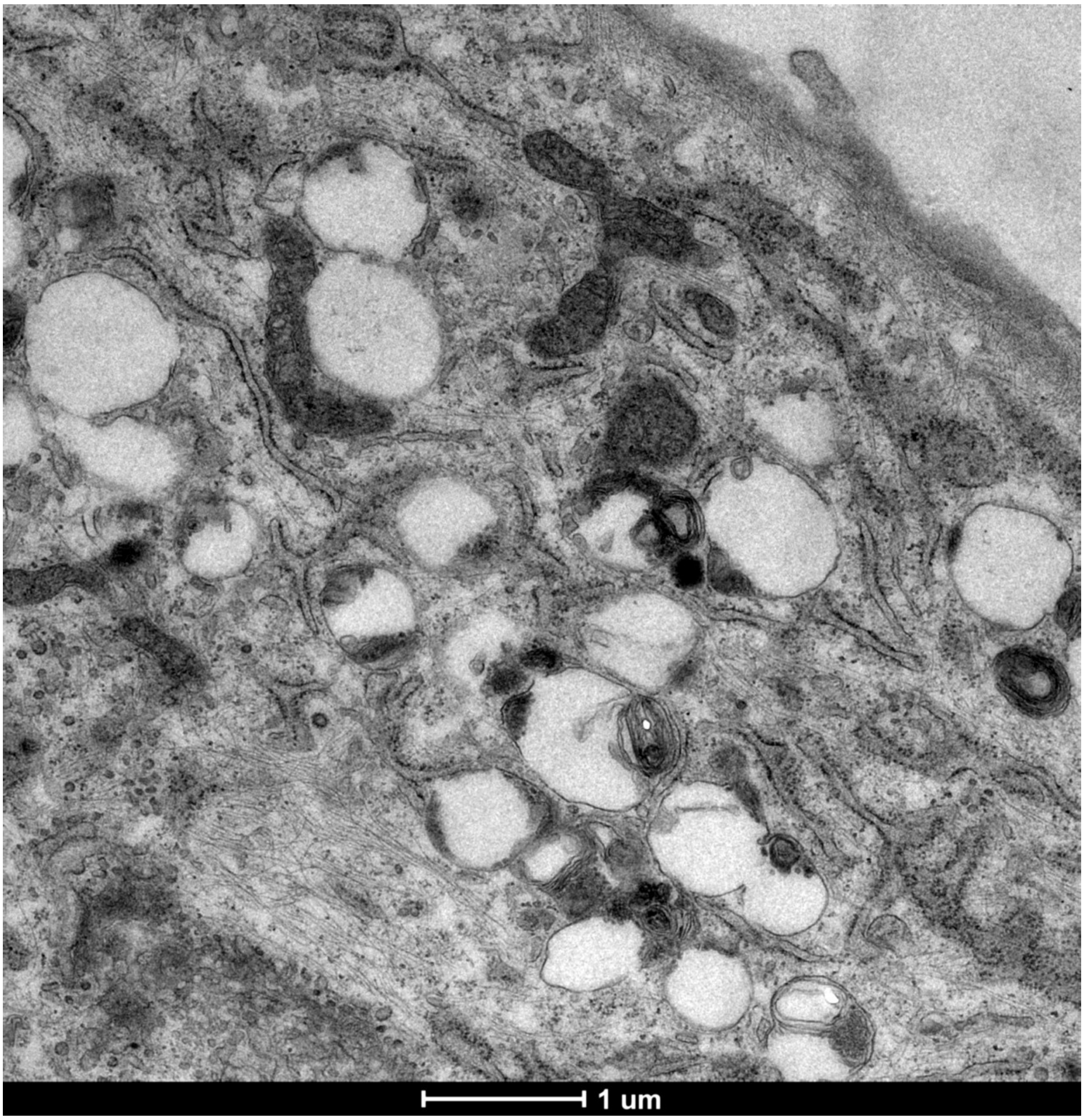

Figure S11. TEM image of FTD-GRN patient primary human fibroblast 03. Scale bar: 1 μm.

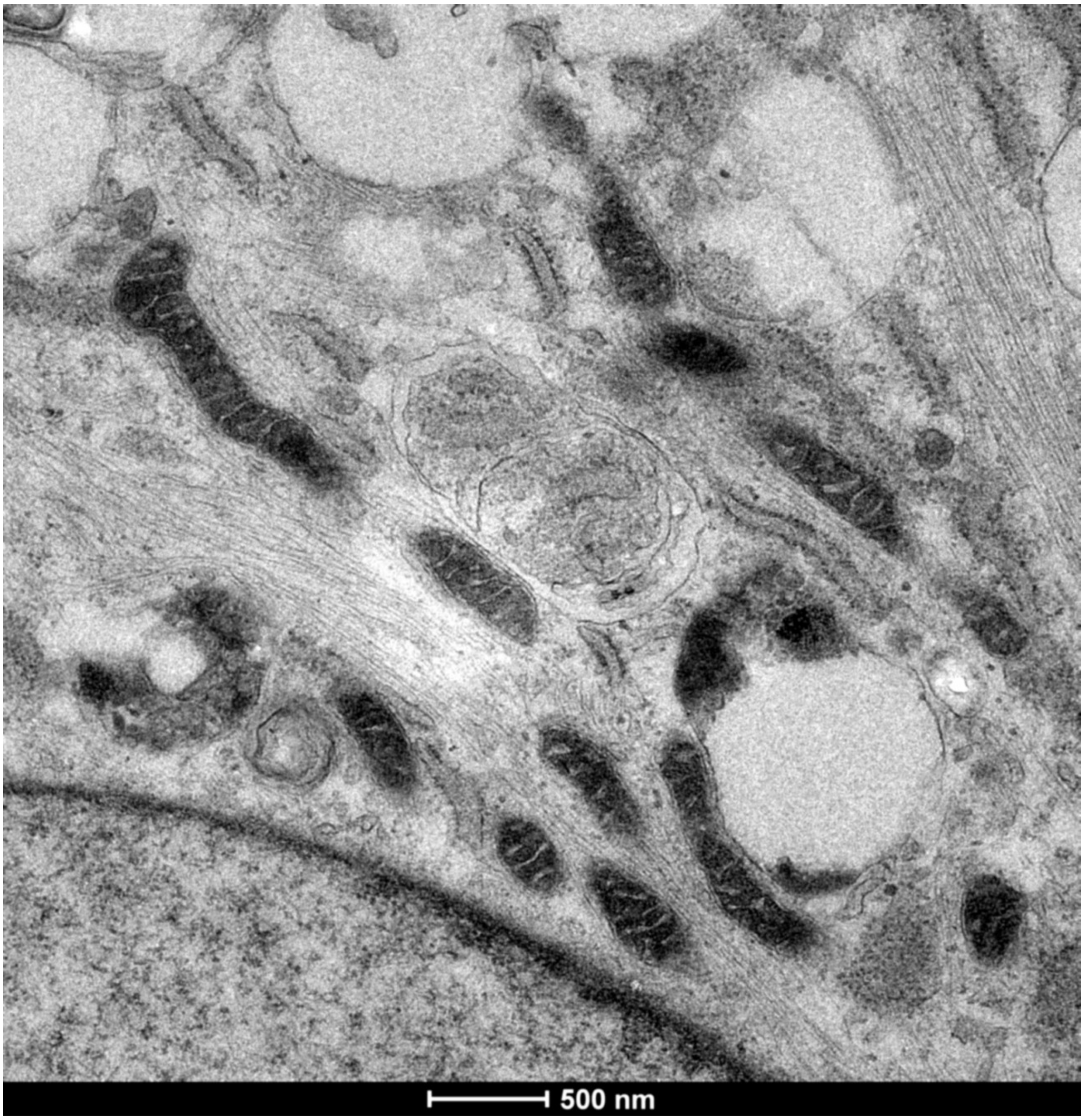

Figure S12. TEM image of healthy control primary human fibroblast 03. Scale bar: 500 nm.

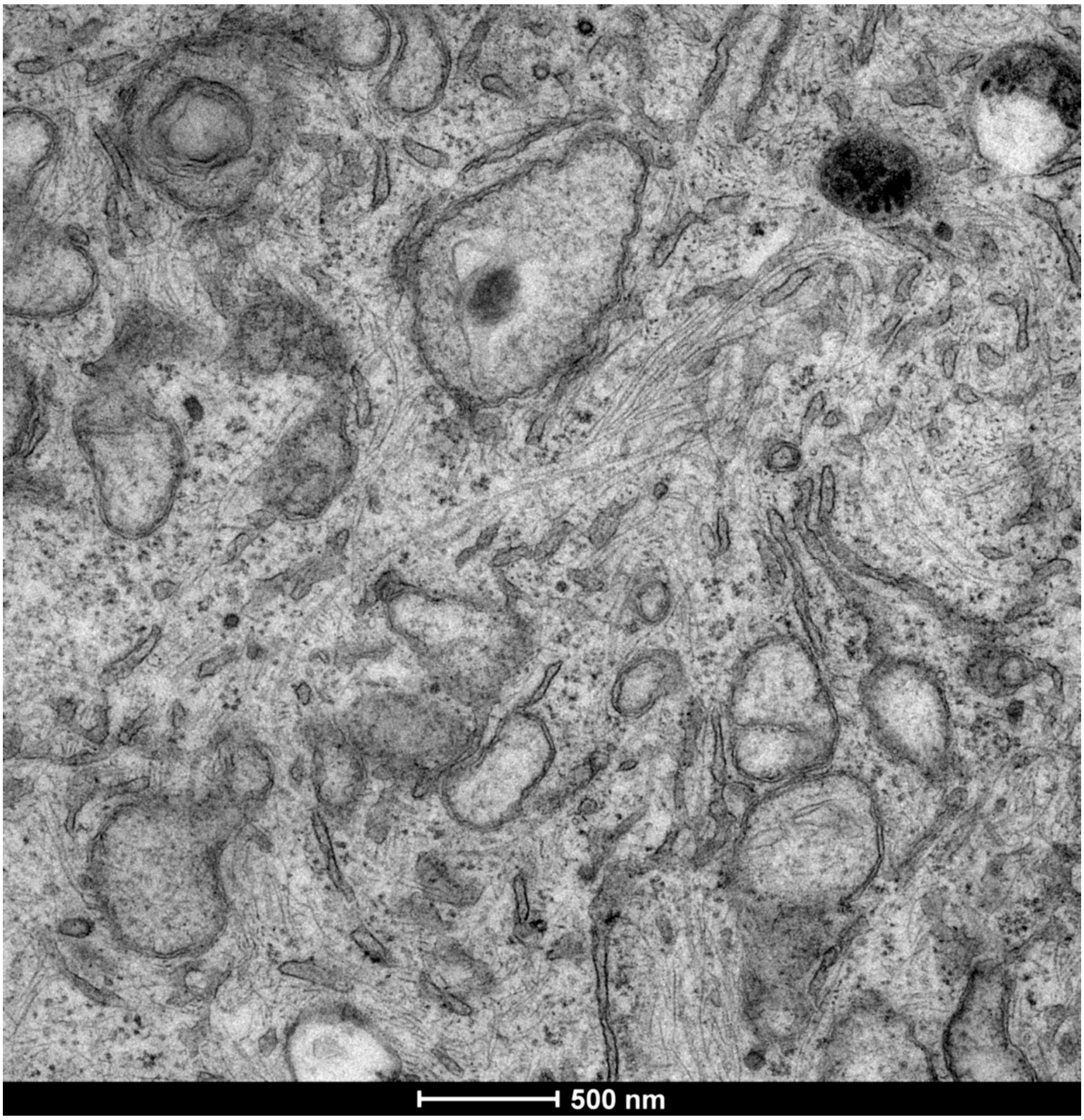

Figure S13. TEM image of FTD-GRN patient primary human fibroblast 04. Scale bar: 500 nm.

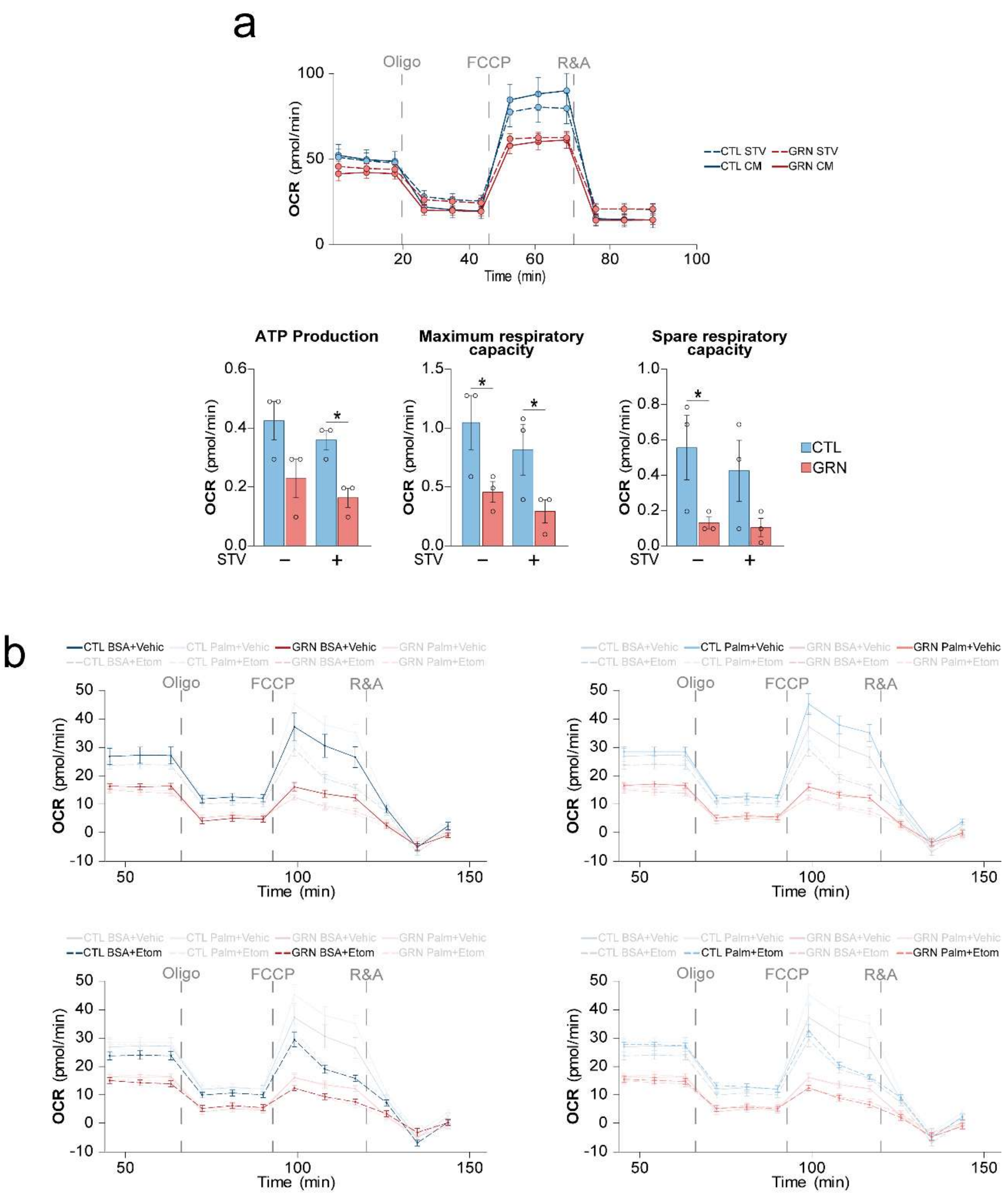

Figure S14. Extension of Figure 4. **a.** Extension of Figure 4c. Seahorse assay depicting mitochondrial oxygen consumption in primary human fibroblasts, with or without a 6-hour starvation (STV) pre-assay period. **b.** Detailed view of Figure 4g. Healthy control fibroblasts (CTL), FTD-GRN patient fibroblasts (GRN), oligomycin (Oligo), rotenone and antimycin (R&A), starving (STV), cell medium (CM), bovine serum albumin (BSA), vehicle (Vehic), palmitic acid (Palm), etomoxir (Etom). Data are presented as mean ± SEM (n = 3).

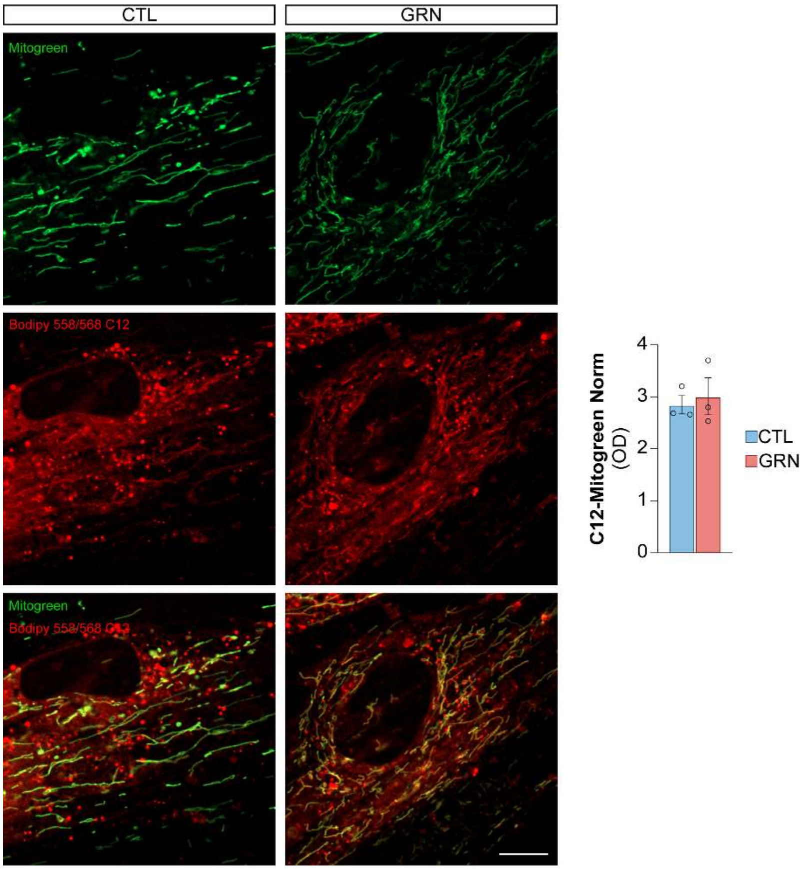

Figure S15. Fatty Acid Localization Inside Mitochondria. **a.** Representative images of Mitogreen (green) and TOM20 (red) staining in primary human fibroblasts. Scale bar: 10 µm. Healthy control fibroblasts (CTL), FTD-GRN patient fibroblasts (GRN). Data are presented as mean ± SEM (n = 3).

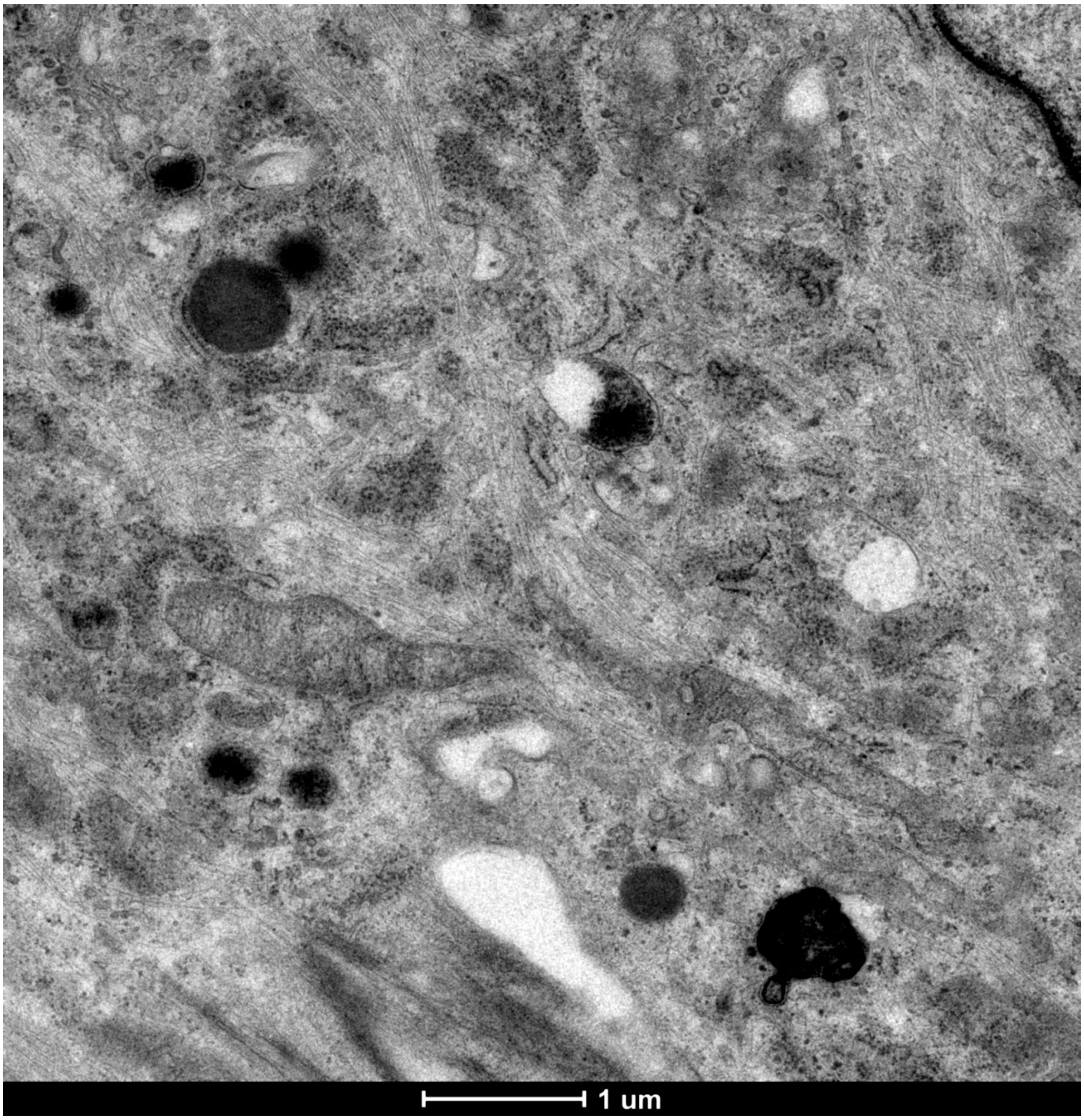

Figure S16. TEM image of healthy control primary human fibroblast 04. Scale bar: 1 μm.

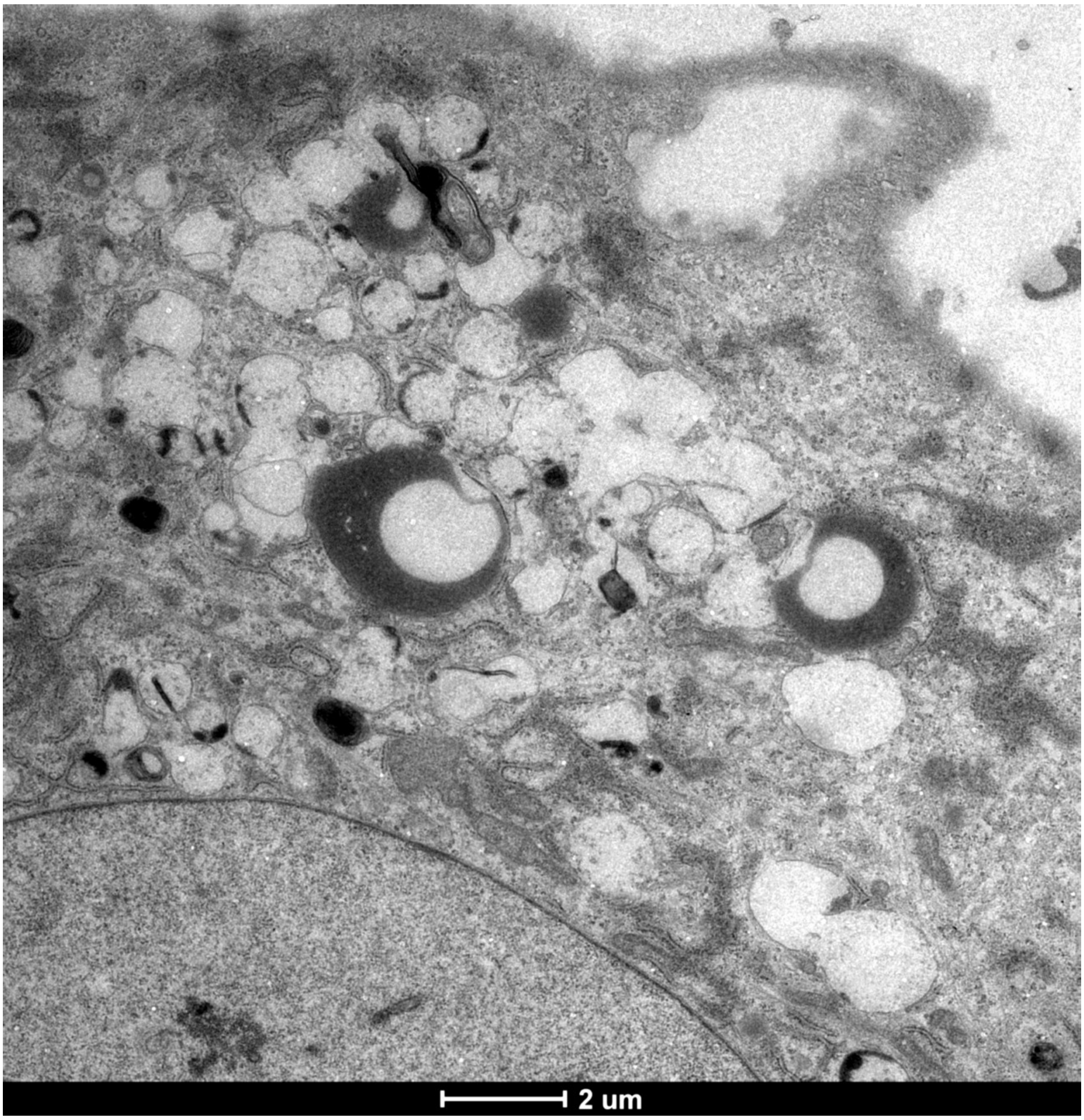

Figure S17. TEM image of FTD-GRN patient primary human fibroblast 05. Scale bar: 2 μm.

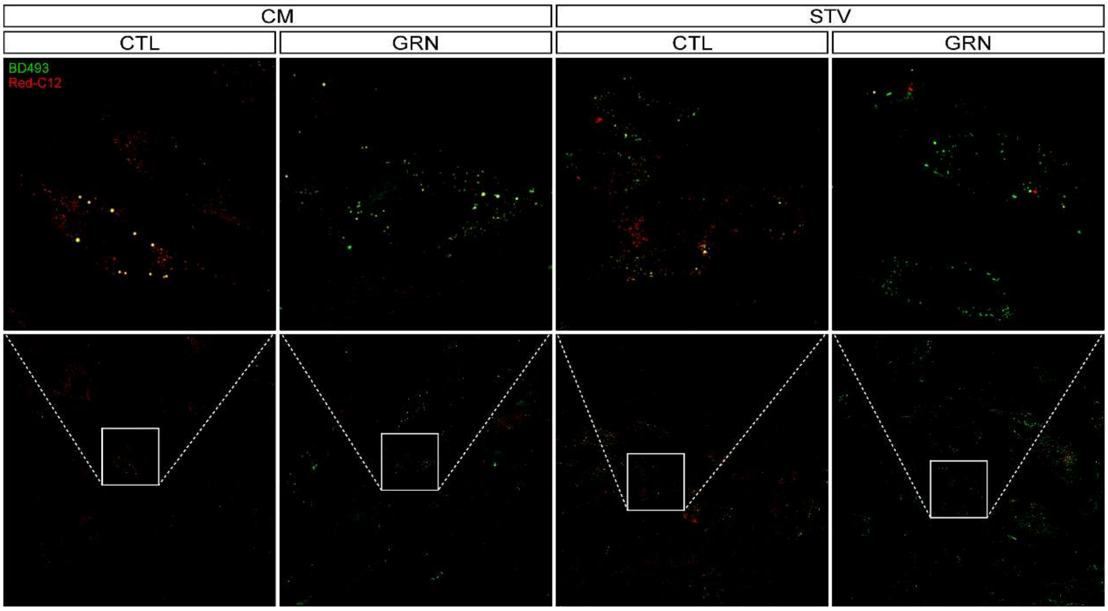

Figure S18. Uncropped Immunofluorescence Images of Figure 5c.

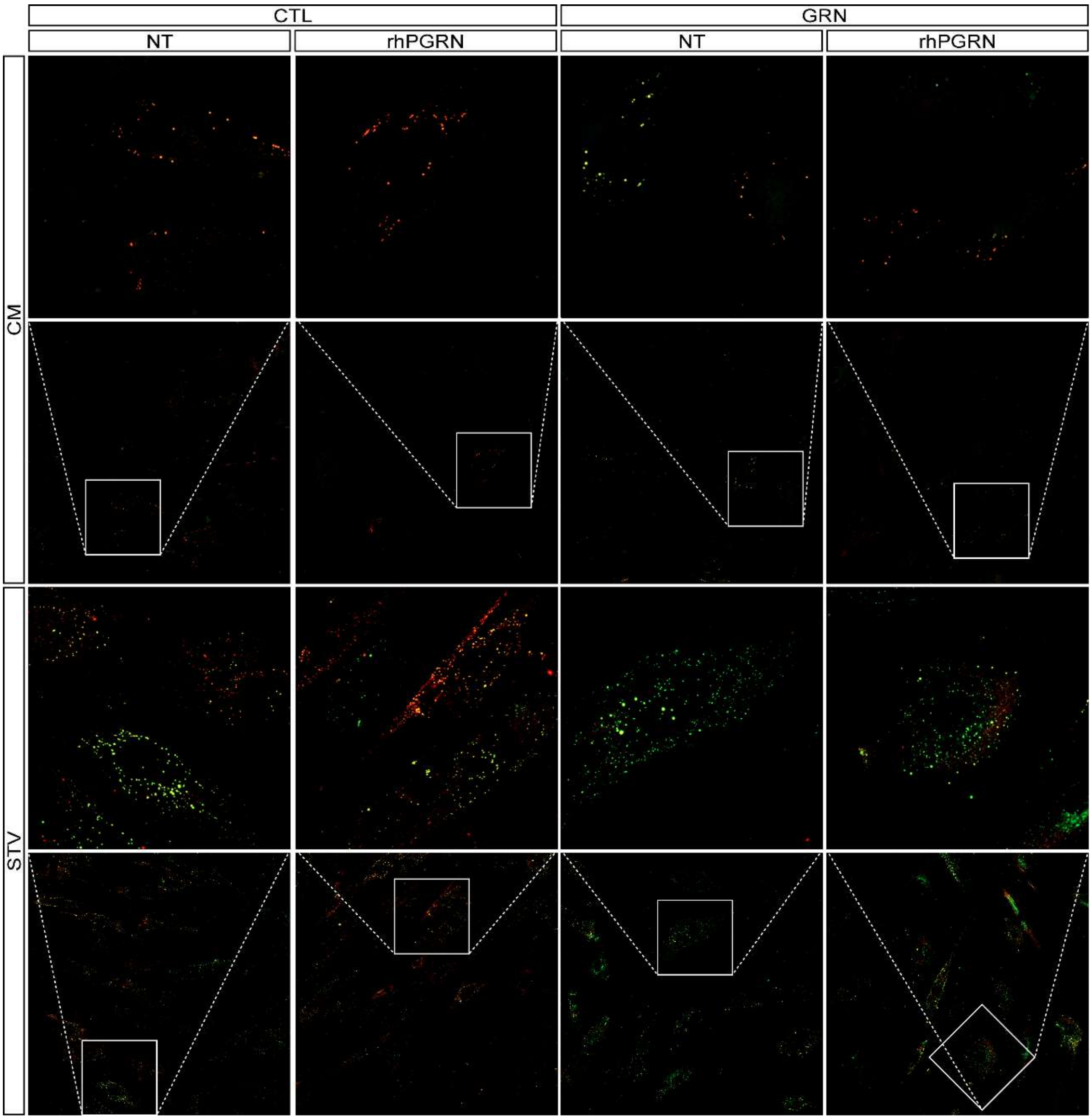

Figure S19. Uncropped Immunofluorescence Images of Figure 5d.
